# IER3 Promotes Malignant Progression of Colorectal Cancer Through the NF‐*κ*B Pathway

**DOI:** 10.1155/ijog/8379666

**Published:** 2026-01-30

**Authors:** Zhigang Wei, Yinyi Luo, Yupeng Zhang, Qingxing Huang, Jianhang Shao, Yingying Zhang, Yuqi Zhang, Zhimin Wang, Chaojie Liang, Zhiyong Lai, Yongping Cui

**Affiliations:** ^1^ Department of Pathology, School of Basic Medical Science, Shanxi Medical University, Taiyuan, China, sxmu.edu.cn; ^2^ Department of Biliary and Pancreatic Surgery, The First Hospital, Shanxi Medical University, Taiyuan, China, sxmu.edu.cn; ^3^ The First Clinical Medical School, Shanxi Medical University, Taiyuan, China, sxmu.edu.cn; ^4^ Department of Colorectal Surgery, The First Hospital, Shanxi Medical University, Taiyuan, China, sxmu.edu.cn; ^5^ Key Laboratory of Cellular Physiology, Ministry of Education, Shanxi Medical University, Taiyuan, China, sxmu.edu.cn

**Keywords:** colorectal cancer, immune checkpoint blockade, immune escape, immunosuppression, tumor immune microenvironment

## Abstract

Colorectal cancer (CRC) remains a leading cause of cancer‐related mortality worldwide, primarily due to metastatic progression and an immunosuppressive tumor immune microenvironment (TIME). The stress‐responsive gene IER3 is found to be dysregulated in multiple cancers. Currently, its functional role in CRC pathogenesis and immune modulation remains poorly understood. Here, using integrated single‐cell RNA sequencing (scRNA‐seq) of clinical samples, we identify a distinct IER3‐expressing malignant subpopulation associated with aggressive disease and poor prognosis. Functional studies demonstrate that IER3 drives CRC proliferation, invasion, and metastatic capacity both *in vitro* and *in vivo*. Mechanistically, IER3 activates the TNF‐*α*/NF‐*κ*B signaling pathway, thereby enhancing the expression and phosphorylation of RELA/p65. Moreover, IER3^+^ malignant cells reshape the TIME into an immunosuppressive state by altering immune cell infiltration and promoting communication via the FN1–CD44 axis. High IER3 expression correlates with reduced response to immune checkpoint blockade (ICB) and distinct drug sensitivity profiles. Together, these findings confirm that IER3 is a dual critical mediator of CRC progression and immune escape, highlighting its potential as a therapeutic target and biomarker for personalized treatment strategies.

## 1. Introduction

Colorectal cancer (CRC) is the third most commonly diagnosed malignancy and the second leading cause of cancer‐related mortality worldwide [1, 2]. Its poor prognosis is largely attributed to the high frequency of hepatic metastasis [3, 4]. Thus, elucidating the biological mechanisms driving CRC progression, identifying predictive biomarkers, and discovering novel therapeutic targets remain major challenges in current research.

Accumulating evidence indicates that the tumor immune microenvironment (TIME) of CRC is markedly immunosuppressive, characterized by an expansion of immunosuppressive cell populations accompanied by reduced infiltration and impaired cytotoxicity of CD8^+^ T cells and NK cells, collectively leading to compromised immune surveillance [5–7]. Although these studies have outlined the immunosuppressive features of CRC, the specific malignant subclones responsible for TIME remodeling and the mechanisms involved remain poorly understood

The stress‐responsive gene immediate early response 3 (IER3) is significantly dysregulated across multiple human cancers and exhibits pleiotropic, context‐dependent roles in regulating cell cycle progression, differentiation, and survival [8–10]. However, due to its dualistic functions, the precise contribution of IER3 to tumor progression, immune evasion, and clinical outcomes remains a matter of controversy [10–12]. Emerging single‐cell data suggest an association between IER3^+^ malignant cells and poor prognosis in CRC; however, whether IER3 promotes CRC progression through NF‐*κ*B signaling and/or immunomodulatory mechanisms remains unclear.

To investigate the molecular basis of CRC progression, we performed single‐cell RNA sequencing (scRNA‐seq) on 26 samples from 16 treatment‐naïve patients with CRC undergoing surgical resection (11 normal tissues and 15 primary tumors). Despite the considerable controversy surrounding the biological role of IER3 in CRC, with evidence supporting both tumor‐suppressive and oncogenic functions, this study sought to resolve this discrepancy. Our comprehensive analysis revealed substantial heterogeneity between primary and metastatic lesions, identifying distinct, aggressive malignant subpopulations characterized by IER3 expression. Through rigorous clinical validation and functional assays both in vitro and in vivo, we established IER3 as a critical regulator of CRC proliferation, invasion, and disease progression. Crucially, we discovered that the crosstalk between IER3^+^ malignant cells and immune cells drives the remodeling of the TIME into an immunosuppressive state, thereby facilitating immune escape. Our findings not only confirm the protumorigenic role of IER3 in CRC but also provide a mechanistic basis for its controversial nature by delineating its specific, context‐dependent function within the immune landscape of aggressive disease subsets. This work positions IER3 as a potential therapeutic target and offers novel insights for improving immune checkpoint blockade (ICB) strategies in CRC.

## 2. Materials and Methods

### 2.1. Human Samples and Ethical Approval

Ethical approval for this study was granted by the Ethics Committee of the First Hospital of Shanxi Medical University (Approval No.: [2020] Ethical Review K012), and written consent was obtained from all participants. We procured 15 tissue specimens from four individuals diagnosed with CRC, including matched normal mucosa, polyp tissues, primary carcinomas, and hepatic metastatic lesions. Archived paraffin‐embedded tissue blocks were also retrieved from 84 subjects, including 14 colorectal liver metastases, 84 primary CRC specimens, 26 colorectal polyps, and 36 normal colorectal mucosa samples. Demographic and clinical parameters were systematically documented through retrospective analysis of medical records and structured interviews with the patients.

### 2.2. Single‐Cell Isolation

NovelBio laboratory personnel performed scRNA‐seq experiments. Resected surgical specimens were immediately preserved in MACS Tissue Storage Solution (Miltenyi Biotec, Bergisch Gladbach, Germany), pending downstream processing. Tissue specimens were rinsed with phosphate‐buffered saline (PBS), minced on ice into fragments of approximately 1 mm^3^, and subjected to enzymatic digestion using 2 units/mL collagenase I (Worthington, Columbus, OH, United States) plus 2 units/mL DNase I (Worthington) for 45 min at 37°C under constant agitation. Following digestion, the suspensions were filtered through 70‐*μ*m strainers and centrifuged (300 × *g*, 5 min). The resulting pellet was resuspended in red blood cell lysis buffer (Miltenyi Biotec) for erythrocyte removal. After washing with PBS containing 0.04% bovine serum albumin (BSA), the cells were resuspended in PBS/0.04% BSA and filtered through 35‐*μ*m strainers. Acridine orange/propidium iodide (AO/PI) staining facilitated the assessment of dissociated single‐cell viability using a Countstar fluorescence cell analyzer. The MACS Dead Cell Removal Kit (Miltenyi Biotec) enrichment was subsequently applied to single‐cell suspensions [13].

### 2.3. Single‐Cell Sequencing

scRNA‐seq libraries were constructed using the Chromium Single Cell 3 ^′^ v3 Reagent Kit and a 10x Genomics Chromium Controller Instrument (10x Genomics, Pleasanton, CA, United States) [14]. Briefly, cellular suspensions were adjusted to approximately 1000 cells/*μ*L before loading onto individual channels for the generation of gel beads‐in‐emulsions (GEMs). Following reverse transcription, GEMs underwent dissolution, enabling the purification and amplification of barcoded cDNA. Amplified barcoded cDNA was subsequently fragmented and subjected to A‐tailing, adapter ligation, and PCR amplification. Final library quantitation was performed using the Qubit High‐Sensitivity DNA Assay (Thermo Fisher Scientific, Waltham, MA, United States), whereas size profiles were evaluated using a High‐Sensitivity DNA Chip on the Bioanalyzer 2200 platform (Agilent Technologies, CA, United States). Sequencing of all libraries was performed on an Illumina instrument (Illumina, San Diego, CA, United States) configured for 150‐bp paired‐end reads.

### 2.4. Single‐Cell RNA Data Processing

scRNA‐seq data analysis was performed by NovelBio Co., Ltd. in collaboration with the NovelBrain cloud analysis platform (https://www.novelbrain.com/). Low‐quality reads and adapter sequences were filtered using *fastp* with default parameters to obtain clean data [15]. Reads were aligned to the human genome (GRCh38, Ensembl Version 91) using CellRanger v3.1.0 to generate the feature–barcode matrix. Downsampling analysis was performed based on barcode read counts per cell per sample, resulting in an aggregated matrix. Cells with fewer than 200 expressed genes or mitochondrial unique molecular identifier (UMI) rates exceeding 20% were excluded, and mitochondrial genes were removed from the expression matrix.

The Seurat package (Version 2.3.4, https://satijalab.org/seurat/) was used for normalizing and regressing UMI counts and mitochondrial rates per sample, yielding scaled data. Principal component analysis (PCA) was performed on the Top 2000 highly variable genes, and t‐SNE and UMAP were constructed using the Top 10 principal components. Unsupervised cell clustering was performed using a graph‐based method, and marker genes were identified using the FindAllMarkers function and Wilcoxon rank‐sum test, based on the following criteria: (1) lnFC > 0.25, (2) *p* value < 0.05, and (3) min.pct > 0.1. Clusters of the same cell type were subjected to re‐UMAP analysis, graph‐based clustering, and marker analysis to refine cell type identification.

### 2.5. Copy Number Variation (CNV) Analysis

Large‐scale chromosomal CNV detection was performed using somatic CNV analysis via the inferCNV R package (v0.8.2); epithelial cells from normal colorectal tissue were used as reference. The CNV burden per cell was computed using the root mean square method applied to genome‐wide CNV values [16]. Malignant classification required simultaneous fulfillment of both a CNV score > 0.05 and an intercellular CNV correlation > 0.5.

### 2.6. Gene Ontology (GO) Enrichment Analysis

To clarify the biological relevance of both marker genes and differentially expressed genes, a comprehensive GO enrichment analysis was performed. GO annotations were obtained from three reputable sources: the GO Consortium (http://www.geneontology.org/), NCBI (http://www.ncbi.nlm.nih.gov/), and UniProt (http://www.uniprot.org/). The statistical significance of enriched GO terms was assessed using Fisher’s exact test, followed by false discovery rate (FDR) adjustment to account for multiple hypothesis testing.

### 2.7. Pathway Enrichment Analysis

To identify biologically significant pathways associated with both marker genes and differentially expressed genes, systematic pathway analysis was conducted using the Kyoto Encyclopedia of Genes and Genome (KEGG) database. Enrichment significance for the identified pathways was evaluated using Fisher’s exact test. Statistical thresholds incorporated both raw *p* values and adjustments based on the FDR.

### 2.8. Gene Set Enrichment Analysis (GSEA)

GSEA was conducted using Hallmark gene sets (MSigDB) to detect pathways exhibiting concordant expression changes across a ranked gene list (by log2 fold change). Significant pathways were defined by the normalized enrichment score (NES) with an FDR *q* value < 0.25, following 1000 permutations.

### 2.9. Pseudotemporal Trajectory Analysis

Single‐cell trajectory inference was conducted using Monocle2 (http://cole-trapnell-lab.github.io/monocle-release) with the DDRtree algorithm under default parameters. Prior to analysis, marker genes identified through Seurat clustering were utilized, and raw expression counts were filtered to select the relevant cells. After pseudotemporal ordering, branch expression analysis modeling (BEAM) [17] was applied to identify key genes regulating cell fate decisions at branching points.

### 2.10. Cell Stemness Assessment

Cellular stemness properties were assessed using the CytoTRACE R package (v0.3.3), a computational method that facilitates differentiation state prediction from single‐cell transcriptomic profiles. This approach quantifies developmental potency along cellular trajectories [18].

### 2.11. Molecular Signaling Communication Analysis

A systematic investigation of intercellular signaling networks was conducted via dual complementary methodologies: (1) CellPhoneDB [19], a curated ligand–receptor interaction repository, and (2) CellChat [20], a computational framework specialized in inferring cell–cell communication from scRNA‐seq data. Annotation was performed for proteins categorized as membrane‐bound, secreted, or extracellular across temporal cell clusters. Significant pairwise interactions were determined using interaction scores and Seurat‐normalized expression matrices, with statistical significance defined as *p* < 0.05.

### 2.12. Immune Infiltration Profiling

We systematically characterized the TIME using bulk tumor RNA‐seq data from the TCGA database and four computational algorithms: CIBERSORT (immune infiltration) [21], ESTIMATE (tumor purity/stroma) [22], tumor immune dysfunction and exclusion (TIDE) (immunotherapy prediction) [23], and oncoPredict (drug sensitivity) [24]. This integrated approach enabled the multidimensional evaluation of immunological features across risk cohorts.

### 2.13. Immunohistochemistry

CRC tissues were fixed in 4% paraformaldehyde, embedded in paraffin, and sectioned at a thickness of 5 *μ*m. Sections were pretreated with a 10% goat serum blocking solution, followed by overnight incubation at 4°C with an anti‐IER3 antibody (1:2000). After washing with PBS, horseradish peroxidase–conjugated secondary antibodies (antimouse or antirabbit) were applied for 1 h at room temperature. The paraffin sections were stained with diaminobenzidine (DAB) and counterstained with hematoxylin. Immunohistochemically stained sections were examined using light microscopy.

### 2.14. Cell Lines and Culture Conditions

Human cell lines (NCM460, SW480, SW620, and HCT116) were obtained from the Shanghai Cell Bank of the Chinese Academy of Sciences, whereas the CCD‐18Co fibroblast cell line was acquired from Meisen Biotech (Zhejiang, China). All cell lines were subjected to comprehensive quality authentication, including morphological examination, postthaw viability assessment, isoenzyme profiling, STR genotyping, and rigorous screening for mycoplasma, bacterial, and fungal contaminations. The cells were maintained in Dulbecco’s modified Eagle medium (DMEM) supplemented with 10% fetal bovine serum and 1% penicillin–streptomycin under standard culture conditions (37°C, 5% CO_2_, and 95% humidity).

### 2.15. CCK‐8 Proliferation Assay

Cells were seeded in 96‐well plates at a density of 2 × 10^3^/100 * μ*L and cultured at 37°C with 5% CO_2_. A working solution was prepared by mixing fresh DMEM with CCK‐8 stock solution (1:1). The original medium was aspirated, and 20 *μ*L of CCK‐8 working solution was added to each well, followed by incubation at 37°C in the dark for 2 h. Absorbance was measured at 450 nm using a microplate reader. OD values at the time points of 1, 2, 3, 4, and 5 days were used to plot CCK‐8 curves and compare groups.

### 2.16. Colony Formation Assay

For colony formation assays, tumor cells were plated in 6‐well plates at a density of 1 × 10^3^/mL and maintained under standard conditions (37°C, 5% CO_2_) for 1–2 weeks with periodic medium replacement. Following colony development, the cultures were washed with PBS after medium removal, fixed using 4% paraformaldehyde, and stained with crystal violet for microscopic evaluation.

### 2.17. Transwell Assay

Migration assays involved seeding 1 × 10^5^ cells suspended in serum‐free DMEM within the upper chambers, while the lower chambers contained 600 *μ*L complete medium supplemented with 10% FBS. Following 48 h of incubation under standard conditions (37°C, 5% CO_2_), nonmigrated upper chamber cells were eliminated. The migrated counterparts underwent 4% paraformaldehyde fixation, followed by crystal violet staining and quantification at 100× magnification. For invasion assessment, Matrigel was diluted 1:10 in DMEM and precoated in the upper chambers for 6 h prior to cell seeding. Subsequent procedures mirrored the migration assay protocols.

### 2.18. Subcutaneous Tumor Formation in Nude Mice

HCT116 cells exhibiting elevated tumorigenicity were isolated, and stable transfectants were generated. These cellular populations were subsequently resuspended in Matrigel at a density of 1 × 10^6^ cells per injection and subcutaneously administered into the flanks of immunodeficient nude mice, with cohorts consisting of six animals each. This investigation utilized 4–5‐week‐old BALB/c nude mice. Tumor dimensions were assessed every other day; volumetric analysis was performed using the following equation: TV (mm^3^) = [length (mm) × width^2^ (mm^2^)]/2. Upon completion of the 6‐week experimental period, subjects underwent euthanasia, permitting excision and gravimetric measurement of the resulting subcutaneous neoplasms.

### 2.19. Preparation of Conditioned Medium

Cells were plated in 6‐well dishes at 5 × 10^5^ density and cultured until they reached 80% confluency. Following replacement with fresh medium containing reduced serum (2% FBS), cultures were maintained for 72 h. Supernatants were subsequently harvested, subjected to centrifugation (1000 × *g*, 5 min) to pellet debris, filtered using 0.22‐*μ*m membranes, and stored at −80°C for subsequent analysis.

### 2.20. Enzyme‐Linked Immunosorbent Assay (ELISA)

Target protein quantification in the conditioned media was performed using an ELISA. The samples and serially diluted standards were introduced into antibody‐coated 96‐well plates, followed by incubation at ambient temperature or 37°C for 60 min. After thorough washing, the plates were incubated with biotin‐conjugated detection antibodies for 60 min. Following subsequent washes, streptavidin–HRP conjugates were applied for 30–60 min. After washing, the TMB substrate was added for chromogenic development (15–30 min, dark conditions). Reactions were terminated with stop solution, and optical density at 450 nm (OD_450_) was immediately measured. Sample protein concentrations were derived from standard curves using ELISA Calc software. All procedures included technical replicates. Washes were performed using PBS containing 0.05% Tween‐20 (PBST) and repeated 3–5 times per step. Data are presented as mean ± SD (*n* = 3 independent experiments).

### 2.21. Statistical Analysis

In the scRNA‐seq data analysis, comparisons of gene expression or signatures across cell groups were performed using unpaired, two‐tailed Student’s *t*‐tests. Assessment of disparities in cellular composition was performed using unpaired two‐tailed chi‐square testing. All statistical procedures and data visualization were executed using R (Version 4.3.1; R Foundation for Statistical Computing, Vienna, Austria). Statistical analyses for cell‐based and animal investigations were conducted using GraphPad Prism (Version 9.4.0; GraphPad Software, San Diego, CA, United States). Comparisons were performed using two‐sided Student’s *t*‐test or one‐way ANOVA. Results are expressed as mean ± standard deviation. Statistical significance was defined as *p* < 0.05.

## 3. Results

### 3.1. The Association Between IER3 Expression and the Clinical Characteristics of CRC

Pan‐cancer analysis revealed significant upregulation of IER3 expression across multiple cancer types, including CRC (Figure 1a). Initial validation of IER3 overexpression in CRC tumors relative to normal tissues was conducted using the TCGA and GEPIA datasets (Figure 1b,c). Immunohistochemical (IHC) analysis of 120 clinical specimens (36 normal and 84 primary CRC) confirmed markedly elevated IER3 protein levels in primary CRC lesions compared to normal mucosa (Figures 1d, 1e, and 1f). High IER3 expression was significantly associated with adverse clinicopathological features, including M1 stage, perineural invasion, poor differentiation, and elevated serum carcinoembryonic antigen (CEA) levels (Figures 1g, 1h, 1i, and 1j; Supporting Information 1: Table S1). Critically, Kaplan–Meier survival analysis demonstrated that patients with high IER3 expression had significantly shorter overall survival (Figure 1k). These data indicate that IER3 is a contributing factor to CRC progression, promoting tumor advancement and predicting adverse clinical outcomes.

Figure 1IER3 is associated with poor prognosis of CRC, as validated in clinical samples. (a) The expression levels of IER3 mRNA in 33 types of cancer were compared with those in normal tissues. (b) Box plot of IER3 expression in CRC tissues obtained from the GEPIA database. (c) Box plot of IER3 expression in colon and rectal cancers from the TCGA database. (d) Immunohistochemical images at 100× and 400× magnifications showing darker brown protein expression in epithelial cells, indicating higher IER3 expression. (e) IER3 levels were examined in paired normal and cancerous tissues from individual patients. (f) Histochemical score histogram. (g) Histogram showing the proportion of M stages in the different IER3 protein expression groups. (h) Histogram showing the proportion of nerve infiltration in the different IER3 protein expression groups. (i) Histogram showing the proportion of tumor differentiation types in different IER3 protein expression groups. (j) Histogram showing the serum CEA concentrations of patients in the different IER3 protein expression groups. (k) Kaplan–Meier curve showing the overall survival of 84 patients with CRC classified according to IER3 protein expression. The *p* value was determined using the log‐rank test. The horizontal axis represents the patient’s survival time (in years), and the vertical axis represents the survival rate. The red line represents the survival curve for patients with high IER3 expression, and the blue line represents the survival curve for patients with low IER3 expression.  ^∗^ *p* < 0.05,  ^∗∗^ *p* < 0.01,  ^∗∗∗^ *p* < 0.001, and  ^∗∗∗∗^ *p* < 0.0001.

**Figure:**
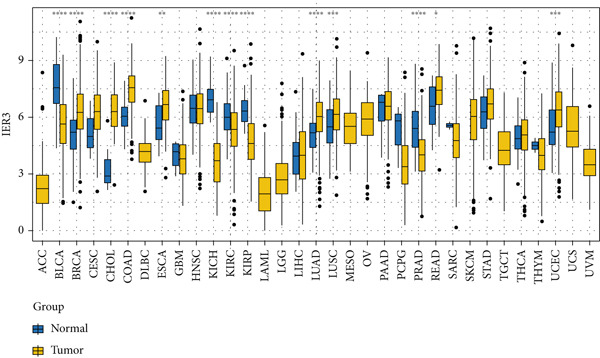
(a)

**Figure:**
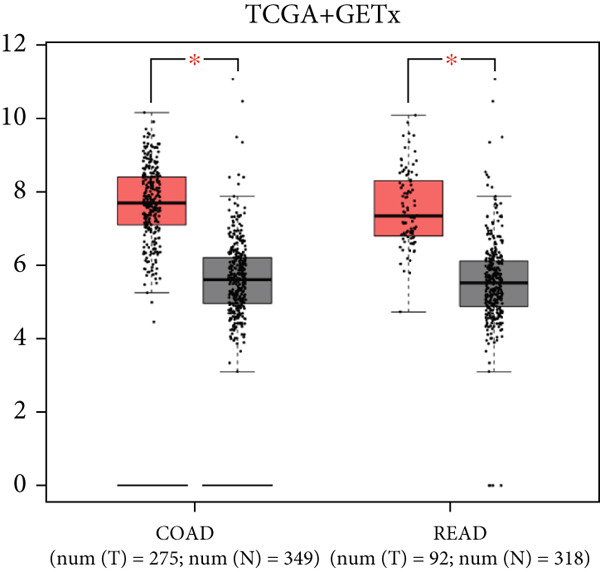
(b)

**Figure:**
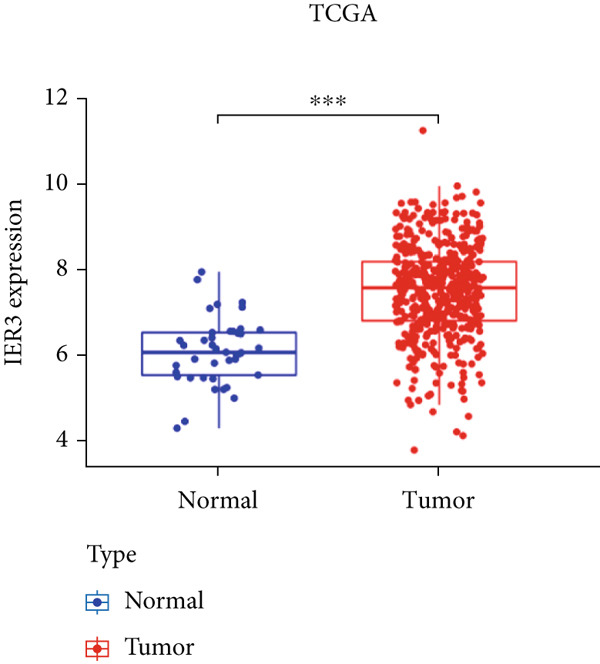
(c)

**Figure:**
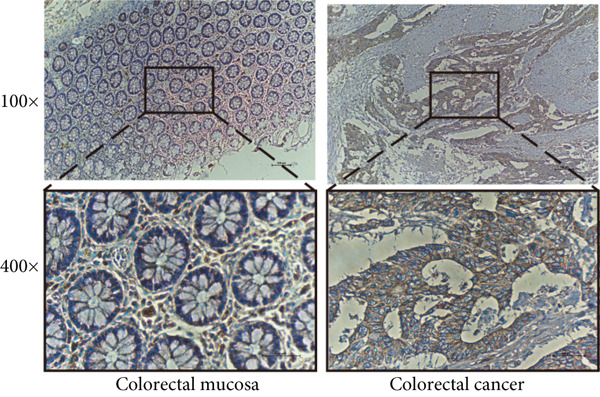
(d)

**Figure:**
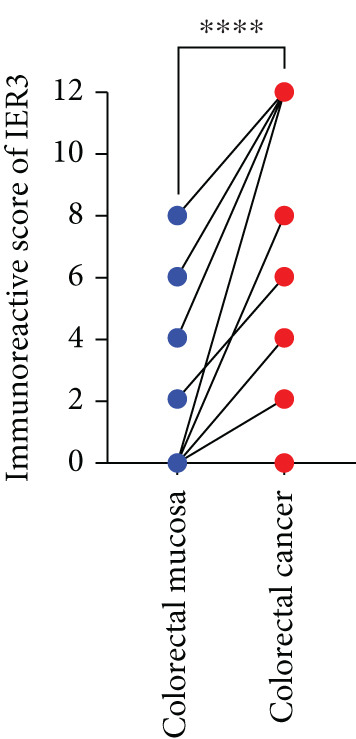
(e)

**Figure:**
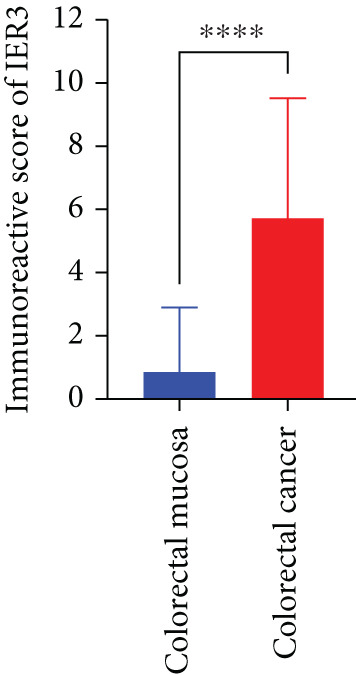
(f)

**Figure:**
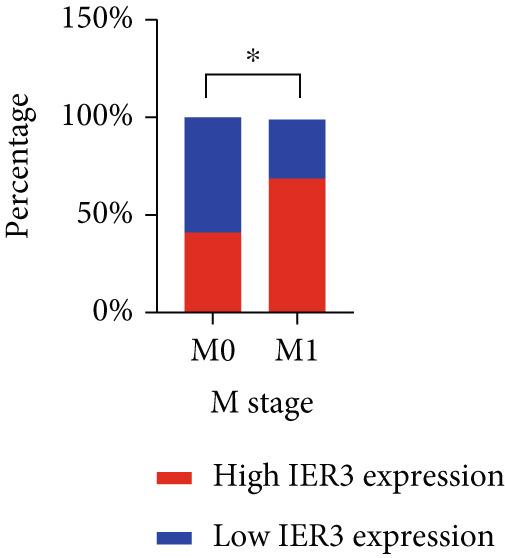
(g)

**Figure:**
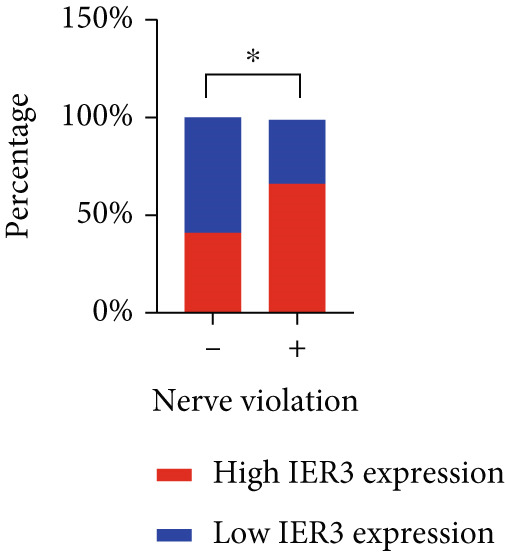
(h)

**Figure:**
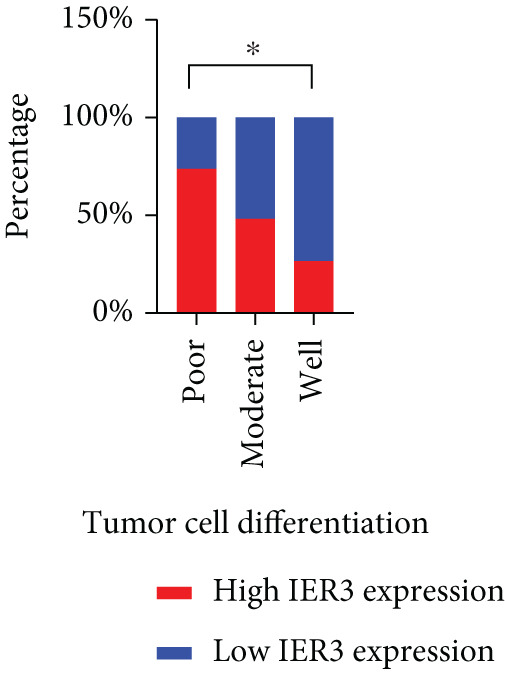
(i)

**Figure:**
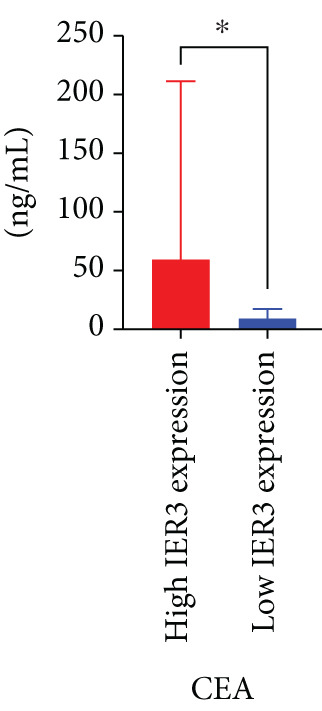
(j)

**Figure:**
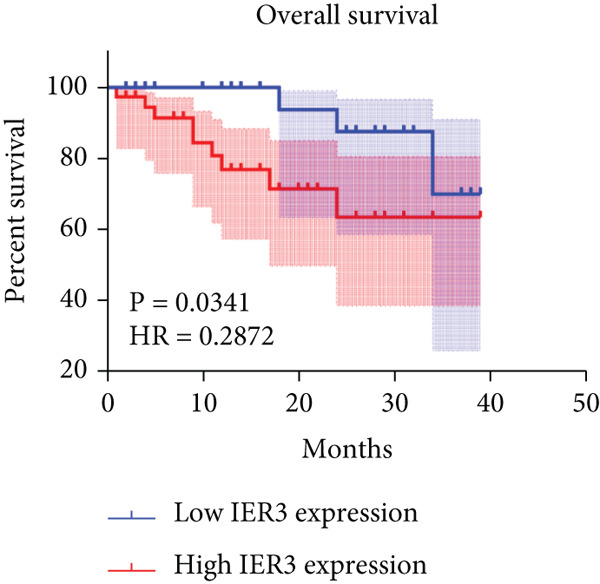
(k)

### 3.2. Identification of an IER3‐Expressing Malignant Cell Subtype Associated With Poor Prognosis in CRC at Single‐Cell Resolution

To characterize CRC at single‐cell resolution, we performed scRNA‐seq on samples collected from 16 treatment‐naïve patients with CRC (Figure 2a). The dataset comprised 11 normal colonic mucosa and 15 primary tumor specimens; the UMAP diagram illustrates the sample source and quality control procedures (Supporting Information 2: Figure S1a,b). After quality control filtering (mitochondrial gene content < 0.4*%*; 200–10,000 genes per cell), a total of 78,377 high‐quality cells from 26 samples were retained for downstream analysis. Unsupervised clustering based on PCA identified 28 major clusters, which were visualized using uniform manifold approximation and projection (UMAP) (Supporting Information 2: Figure S1c).

Figure 2Identification of IER3 malignant cell subtypes at single‐cell resolution is associated with poor prognosis of CRC. (a) Schematic diagram of the single‐cell sequencing process in the experiment. (b) UMAP visualizes different cell types, each represented by a different color. (c) The bubble chart depicts the expression levels of typical marker genes in the major cell types. The size of the dot indicates the proportion of cells expressing a gene in the cell type cluster. The dot color indicates the average scaled expression level of this gene in the cells expressed in this cluster. (d) UMAP visualization of epithelial cells, colored to distinguish malignant cells (defined by the CNV threshold) from nonmalignant cells. (e) The bubble chart depicts the expression levels of typical marker genes in epithelial cell types. (f) UMAP visualizes different types of epithelial cells, each represented by a different color. (g) UMAP visualization of IER3 expression in epithelial cells. (h) The violin diagram illustrates the differences in IER3 expression across various tissues. (i) UMAP visualizes different cell types, each represented by a different color. (j) The violin plot represents the differences in cnv_ among different cell subpopulations. (k) The Kaplan–Meier curve illustrates the overall survival of 610 patients with CRC from TCGA cohort, categorized by the expression of the IER3_High_malignant characteristic gene. The *p* value was determined using the log‐rank test.  ^∗^ *p* < 0.05,  ^∗∗^ *p* < 0.01,  ^∗∗∗^ *p* < 0.001, and  ^∗∗∗∗^ *p* < 0.0001.

**Figure:**
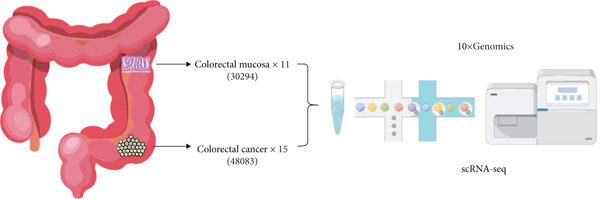
(a)

**Figure:**
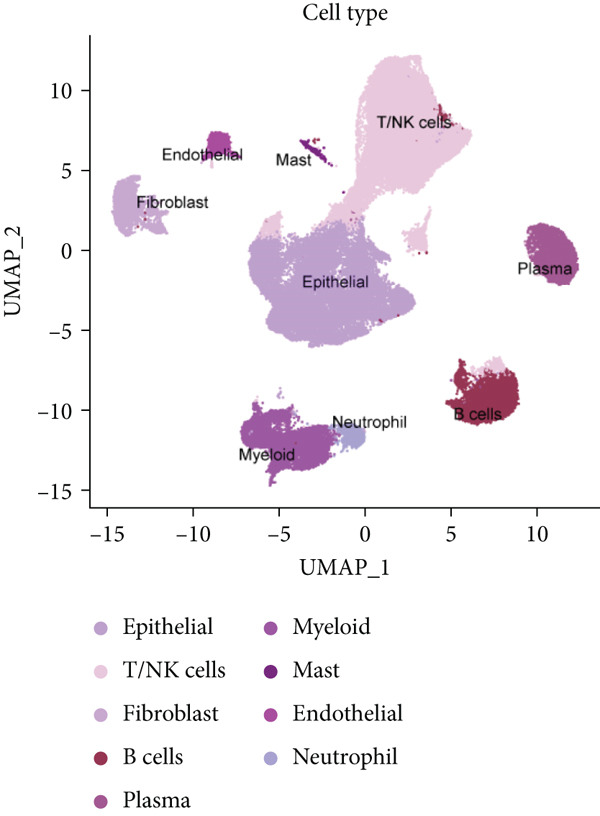
(b)

**Figure:**
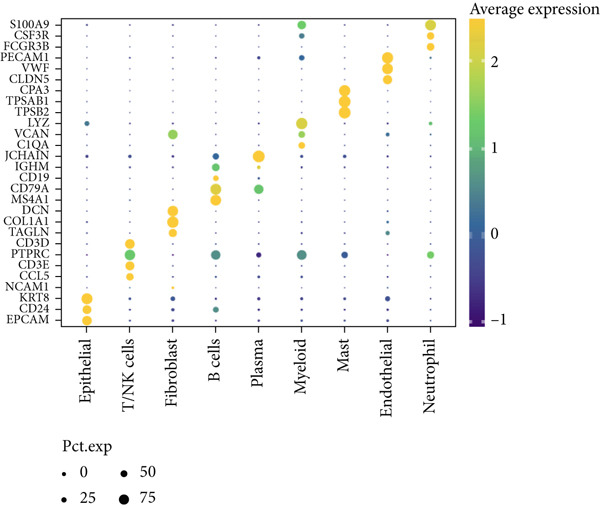
(c)

**Figure:**
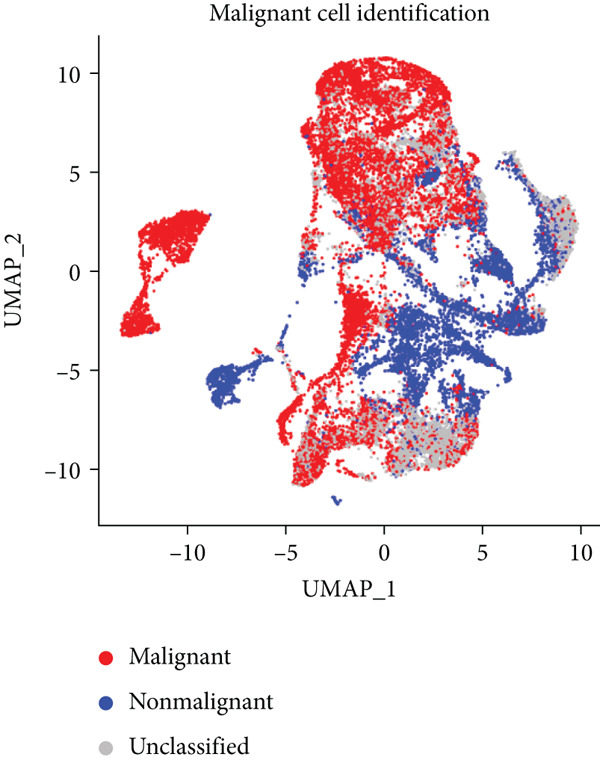
(d)

**Figure:**
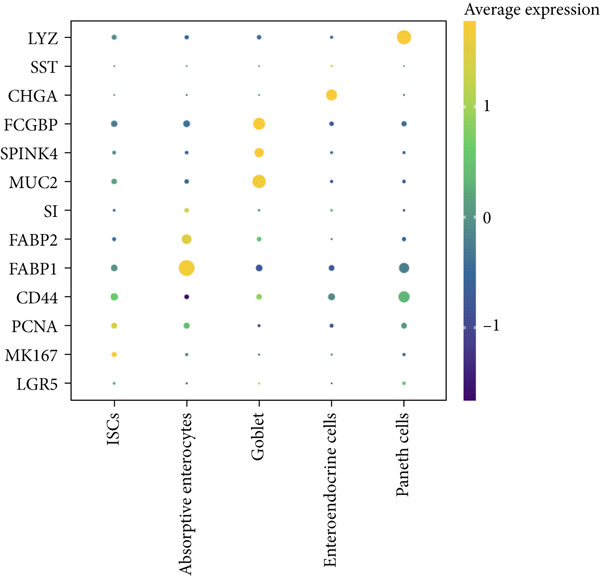
(e)

**Figure:**
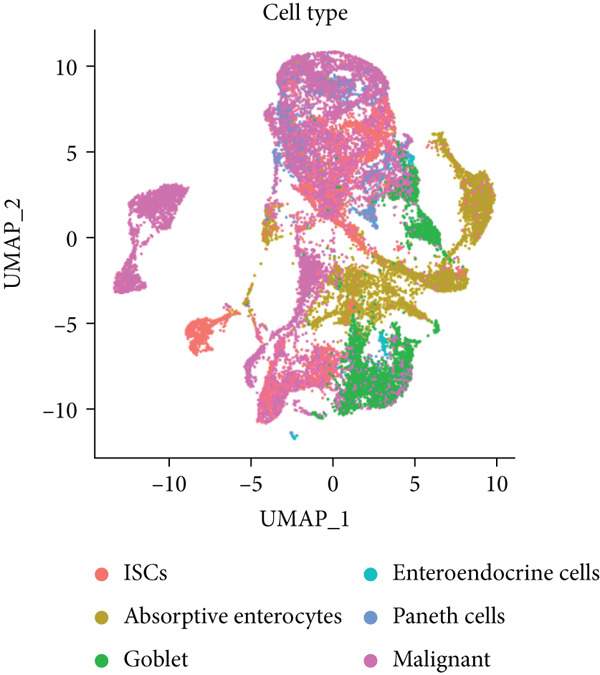
(f)

**Figure:**
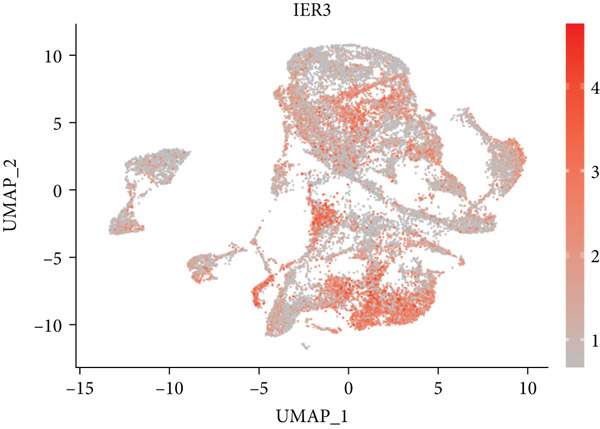
(g)

**Figure:**
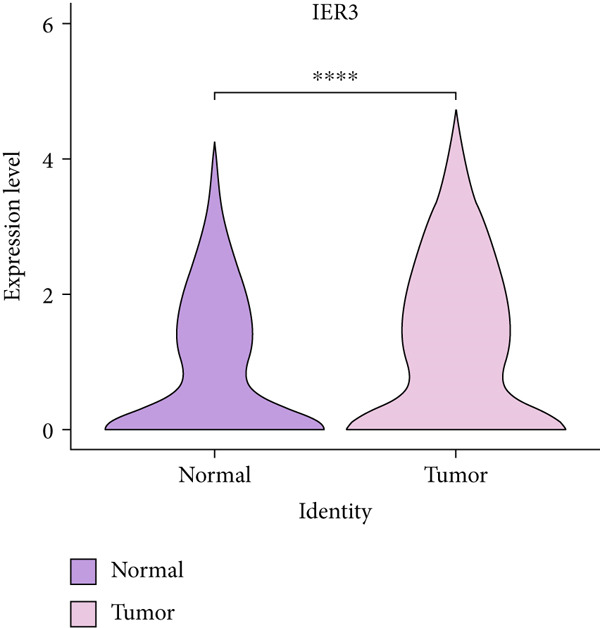
(h)

**Figure:**
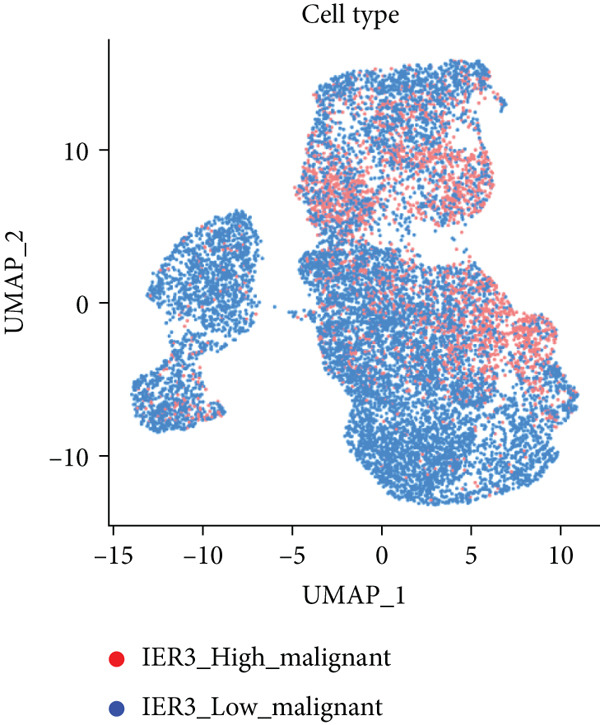
(i)

**Figure:**
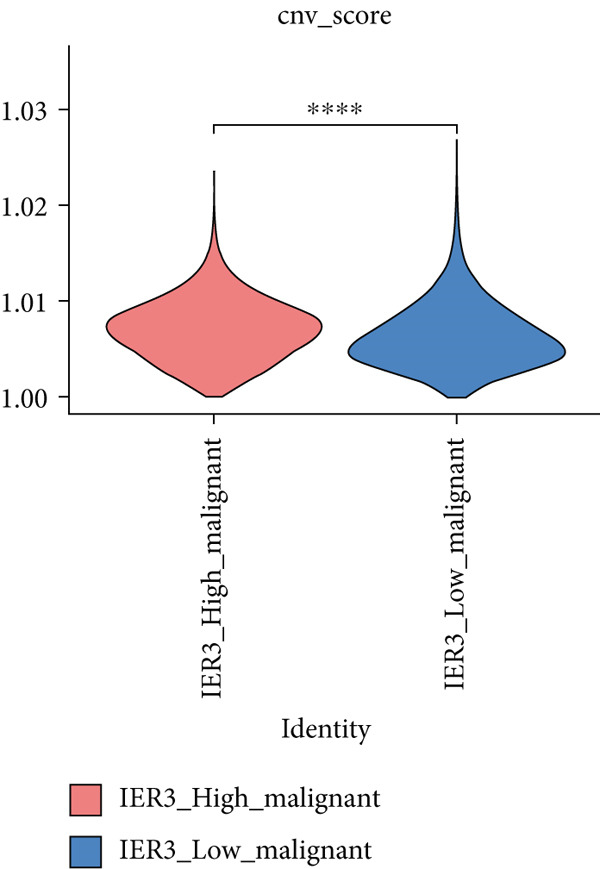
(j)

**Figure:**
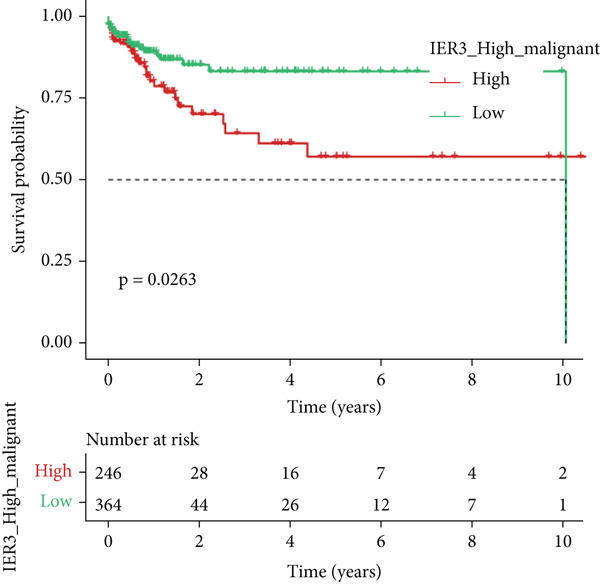
(k)

Cells were annotated into 10 distinct lineages according to the established marker genes: T/NK cells (CCL5, CD3E, CD3D, and PTPRC), B cells (MS4A1, CD79A, and CD19), plasma cells (IGHM and JCHAIN), myeloid cells (C1QA, VCAN, and LYZ), neutrophils (FCGR3B, CSF3R, and S100A9), mast cells (TPSB2, TPSAB1, and CPA3), endothelial cells (CLDN5, VWF, and PECAM1), epithelial cells (KRT8, CD24, and EPCAM), and fibroblasts (TAGLN, COL1A1, and DCN) (Figure 2b,c). A comparative analysis revealed a reduced abundance of B lymphocytes and plasma cells in tumor tissues compared to those in normal mucosa. In contrast, neutrophils, myeloid cells, and endothelial cells were significantly enriched in CRC samples, underscoring the remodeling of the tumor microenvironment (TME) during malignant progression (Supporting Information 2: Figure S1d,e).

Given their pivotal role in tumor initiation and progression, epithelial cells were subjected to further subclustering (Supporting Information 2: Figure S1f,g). Putative malignant cells were identified by inferential CNV analysis using normal epithelial cells as a reference (Supporting Information 2: Figure S1h). Malignant epithelial cells were stratified based on CNV profiles and annotated according to cluster‐defined gene expression signatures (Figure 2d,e), yielding six distinct subpopulations that were visualized using UMAP (Figure 2f).

We next evaluated IER3 expression across epithelial subpopulations and observed pronounced upregulation in malignant clusters (Figure 2g). A quantitative comparison confirmed significantly elevated IER3 expression in tumor cells versus normal epithelial cells (Figure 2h). Cells with IER3 expression in the Top 25th percentile were classified as IER3_High_malignant, whereas the remainder were designated as IER3_Low_malignant (Figure 2i). The IER3_High_malignant subgroup demonstrated significantly higher malignant potential than the IER3_Low_malignant subgroup (Figure 2j). Consistently, analysis of TCGA CRC cohort revealed that patients enriched for the IER3_High_malignant subtype exhibited significantly reduced overall survival (Figure 2k and Supporting Information 2: Figure S1i).

### 3.3. Characterization of the IER3‐Expressing Malignant Subtype in CRC

We next used CytoTRACE to deduce the cellular differentiation states (Supporting Information 2: Figure S1j) and applied Monocle 3 to reconstruct the developmental trajectory of malignant epithelial cells (Figure 3a). Trajectory inference suggested progression originating from IER3_Low_malignant cells and advancing toward IER3_High_malignant cells (Figure 3b). We further evaluated the expression dynamics of key genes associated with the IER3_High_malignant subpopulation across pseudotime and observed a progressive increase in IER3, CCL20, and FN1 (Figure 3c).

Figure 3Characteristics of IER3_High_malignant cells. (a) Monocle trajectory inference of malignant cells colored by their corresponding pseudotime. (b) Monocle trajectory inference of malignant cell subpopulations. (c) Dynamic changes in the expression of the key gene IER3_High_malignant along pseudotime. (d) Volcano map showing differentially expressed genes IER3_High_malignant and IER3_Low_malignant, with red indicating upregulation of the gene and green indicating downregulation. (e) GO analysis revealed the main enrichment pathways of differentially expressed genes. (f) KEGG pathway analysis revealed the primary enriched pathways of the differentially expressed genes. (g) Results of GSEA.

**Figure:**
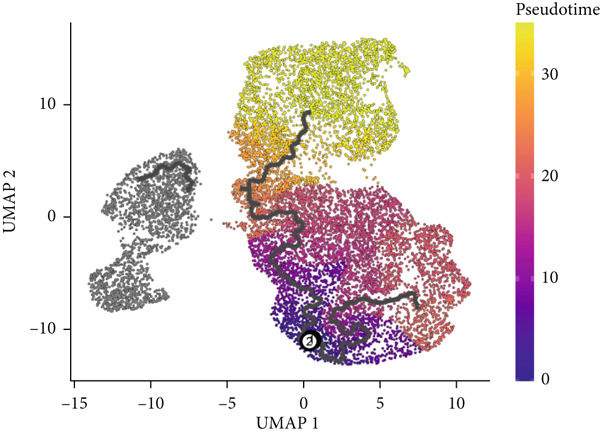
(a)

**Figure:**
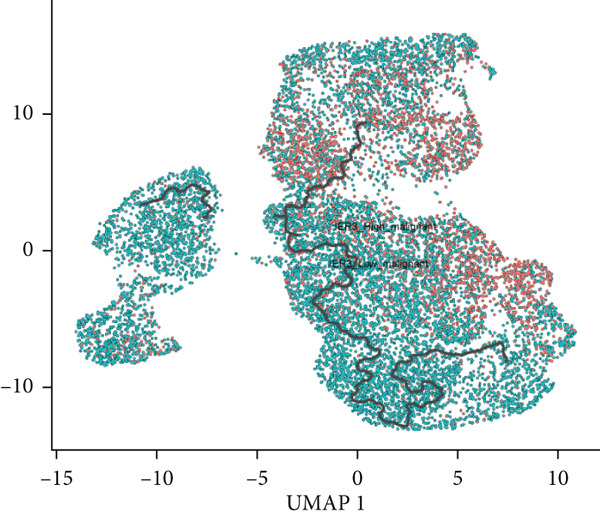
(b)

**Figure:**
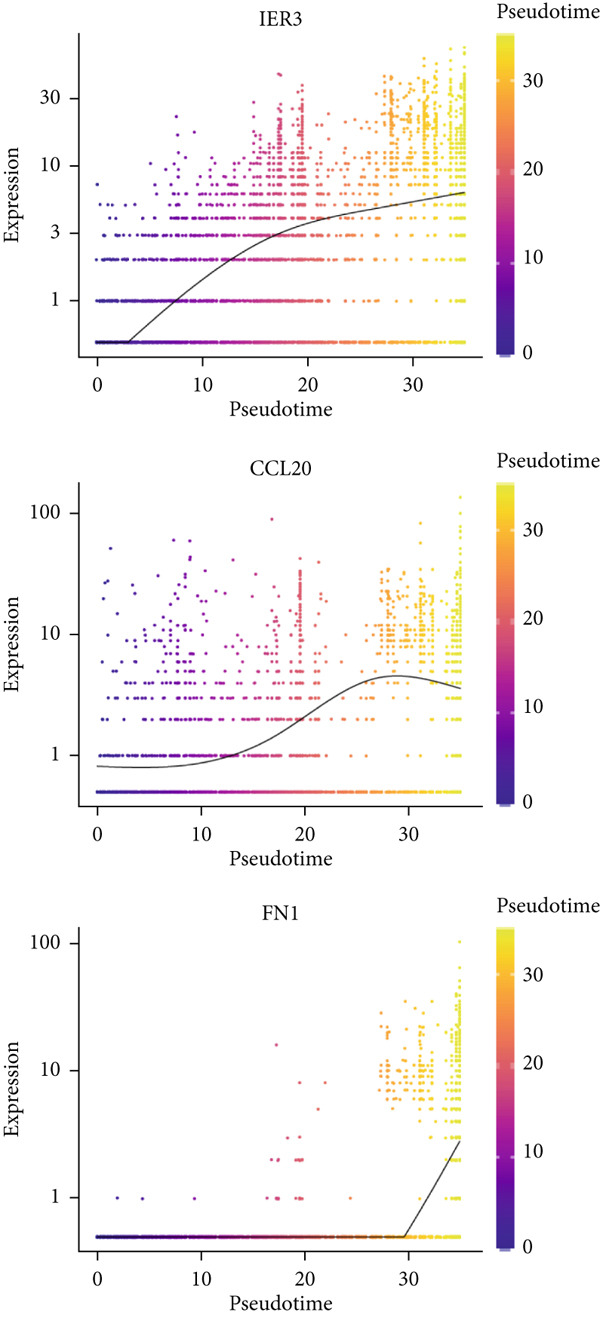
(c)

**Figure:**
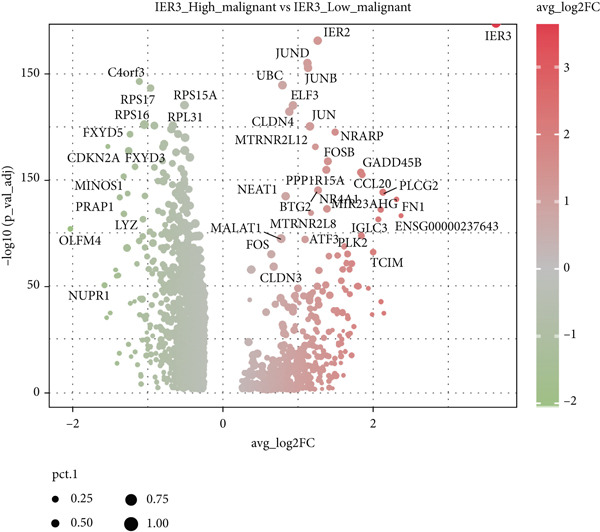
(d)

**Figure:**
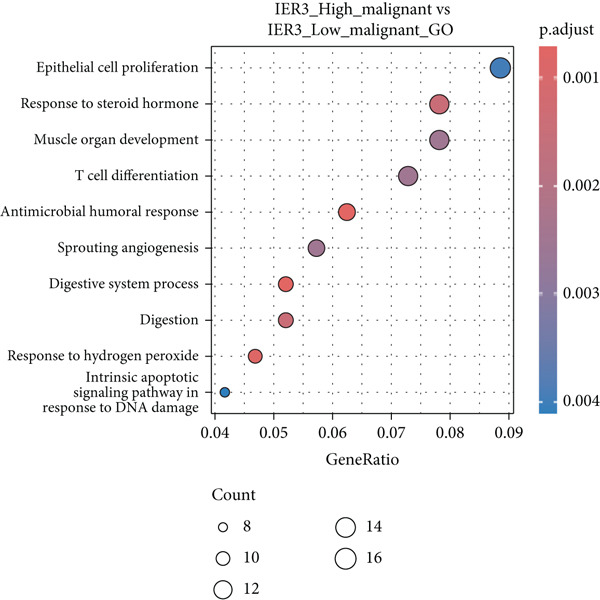
(e)

**Figure:**
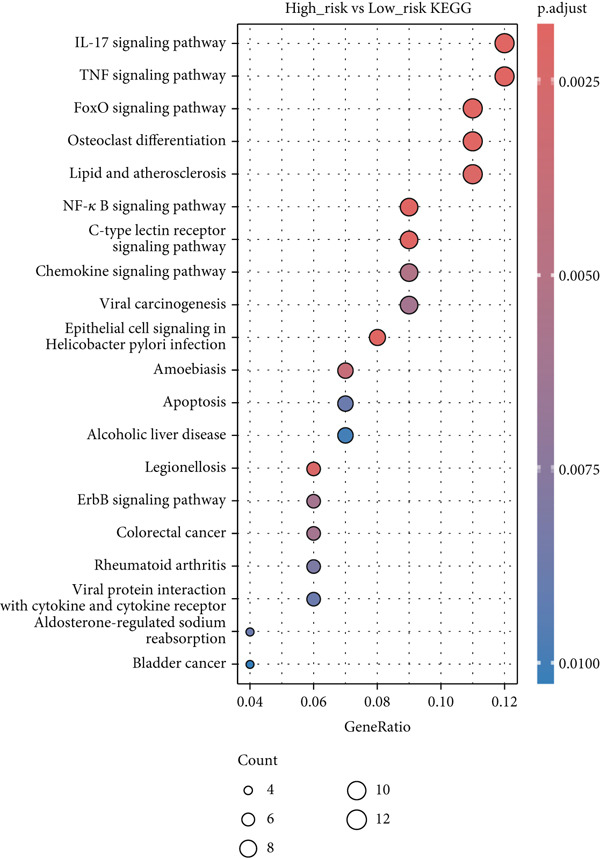
(f)

**Figure:**
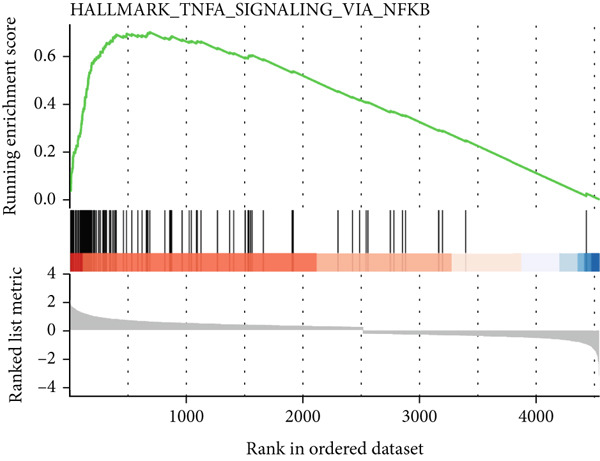
(g)

Differential gene expression analysis identified 487 upregulated and 1523 downregulated genes in IER3_High_malignant cells compared to IER3_Low_malignant cells (Figure 3d). GO enrichment analysis indicated that upregulated genes were significantly enriched in biological processes related to epithelial cell proliferation (Figure 3e). KEGG pathway analysis revealed notable enrichment in several oncogenic signaling pathways, including NF‐*κ*B signaling [25–27], ErbB signaling [28], IL‐17 signaling [29], and TNF signaling [30] (Figure 3f). GSEA further confirmed significant activation of the TNF‐*α*/NF‐*κ*B signaling pathway in IER3_High_malignant cells (Figure 3g). Together, these findings suggest that the IER3_High_malignant subtype facilitates CRC progression through the synergistic dysregulation of immunomodulatory and inflammatory signaling pathways.

### 3.4. IER3 Promotes Tumor Growth and Invasion In Vitro and In Vivo

qPCR analysis revealed significantly elevated IER3 mRNA expression in CRC cell lines (SW480, SW620, and HCT116) compared to that in normal colonic epithelial cells (NCM460) (Figure 4a). Stable IER3‐knockdown and IER3‐overexpressing cell lines were generated in HCT116 and SW480 cells via lentiviral transduction, and efficient modulation was confirmed by immunoblotting (Figure 4b).

Figure 4IER3 promotes tumor cell growth and invasion in vitro and in vivo. (a) PCR results showing IER3 mRNA expression levels in different cell lines. (b) Western blot results showing the expression level of IER3 protein in stably transformed cell lines. (c) Proliferation curves of CRC cells assessed using the CCK‐8 assay. (d) Colony formation assay results and the corresponding bar plot showing colony statistics following IER3 knockdown in CRC cells. (e) Colony formation assay results and the corresponding bar plot showing colony statistics after IER3 overexpression in CRC cells. (f) Transwell assay results showing the migration and invasion of CRC cells after IER3 knockdown, with representative images at 100× magnification and a bar plot quantifying the number of migrating and invasive cells. (g) Transwell assay results showing the migration and invasion of CRC cells after IER3 overexpression, with representative images at 100× magnification and a bar plot quantifying the number of migrating and invasive cells. (h) Results of subcutaneous tumor formation experiments in nude mice using CRC cells overexpressing IER3. (i) Tumor volume growth curves (cubic millimeter) obtained from subcutaneous tumor formation experiments in nude mice with CRC cells overexpressing IER3.  ^∗^ *p* < 0.05,  ^∗∗^ *p* < 0.01,  ^∗∗∗^ *p* < 0.001, and  ^∗∗∗∗^ *p* < 0.0001.

**Figure:**
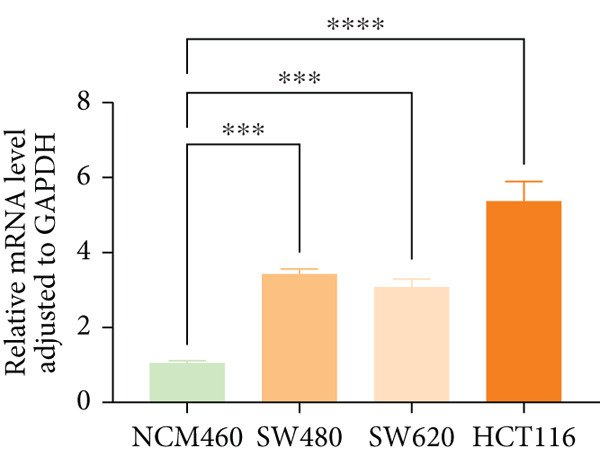
(a)

**Figure:**
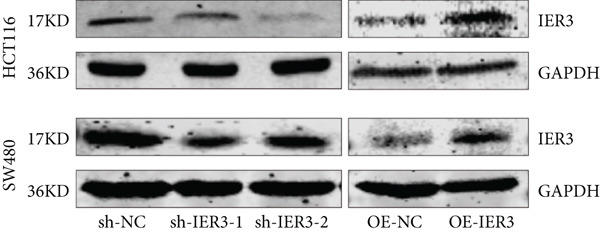
(b)

**Figure:**
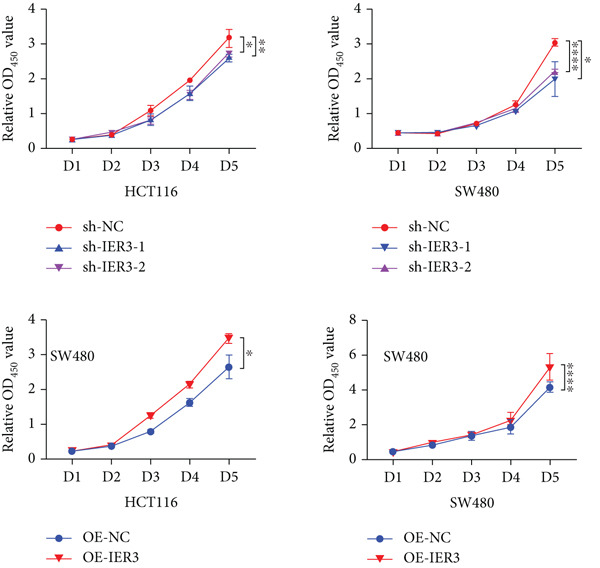
(c)

**Figure:**
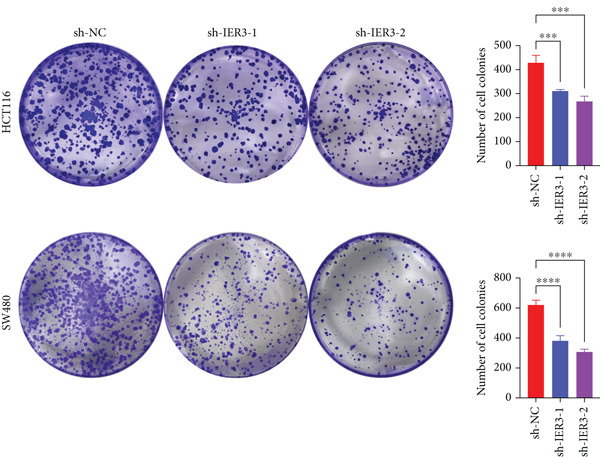
(d)

**Figure:**
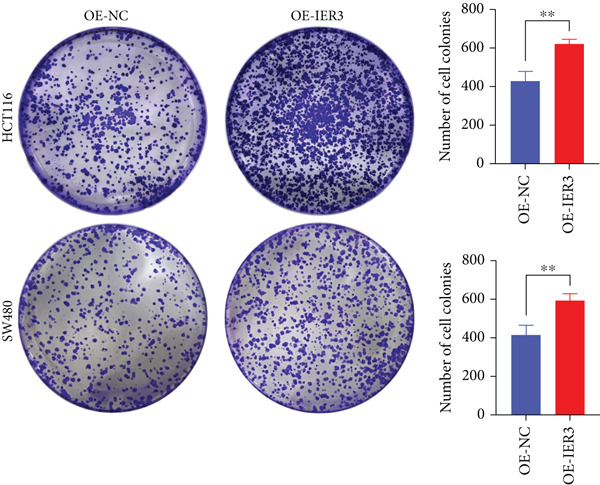
(e)

**Figure:**
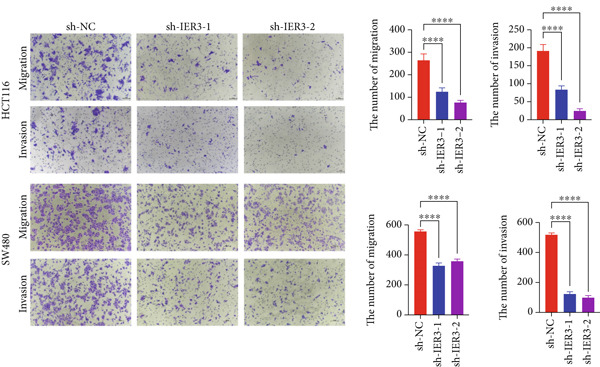
(f)

**Figure:**
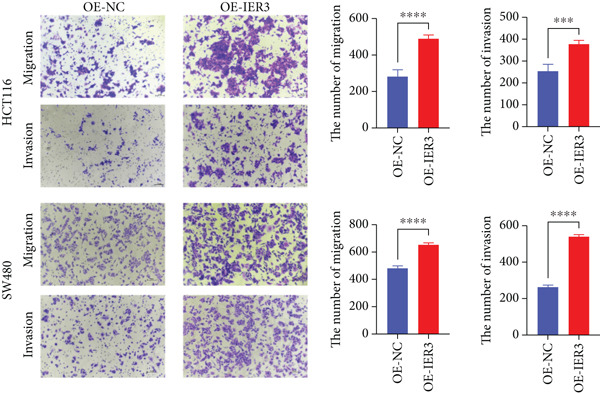
(g)

**Figure:**
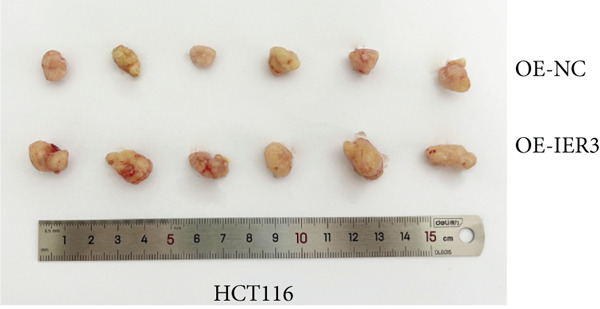
(h)

**Figure:**
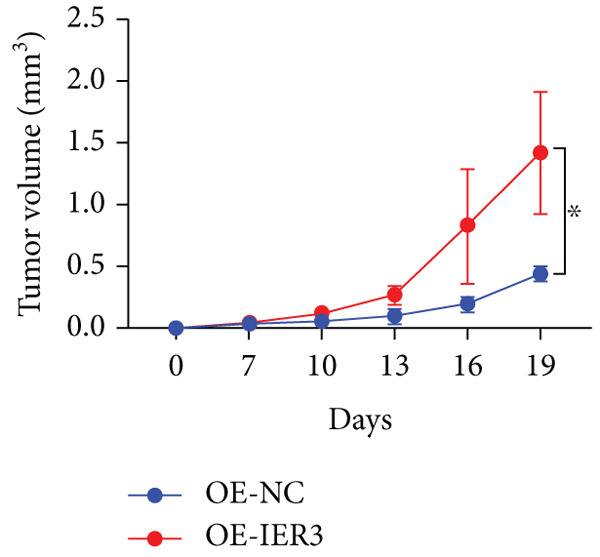
(i)

CCK‐8 assays showed that IER3 knockdown suppressed cell proliferation, whereas IER3 overexpression enhanced cell viability (Figure 4c). Consistent with these findings, colony formation capacity was reduced following IER3 depletion but increased upon IER3 overexpression (Figure 4d,e). Transwell migration and invasion assays further demonstrated that IER3 knockdown impaired migratory and invasive abilities, whereas IER3 overexpression promoted both activities (Figure 4f,g). In vivo, xenograft tumors derived from IER3‐overexpressing cells exhibited accelerated growth and larger tumor volumes, whereas IER3 knockdown resulted in significantly reduced tumorigenicity in nude mice (Figure 4h,i).

These results establish that IER3 promotes metastatic competence by augmenting self‐renewal, providing a mechanistic basis for the predominance of IER3‐positive cells in CRC progression.

### 3.5. IER3 Activates TNF‐*α*/NF‐*κ*B Signaling

Analysis of the TCGA cohort using GSEA demonstrated significant enrichment of the Hallmark TNF‐*α*/NF‐*κ*B signaling pathway in patients with CRC with elevated IER3 expression (Figure 5a). Coexpression analysis revealed significant positive correlations between IER3 levels and the expression of both TNF‐*α* and the RELA/p65 subunit (Figures 5b,c). ELISA showed that IER3 knockdown in tumor cells reduced TNF‐*α* protein secretion, whereas IER3 overexpression markedly increased TNF‐*α* secretion relative to the controls (Figure 5d). Following supplementation of tumor cell cultures with recombinant TNF‐*α* protein to stimulate the NF‐*κ*B pathway exogenously, analysis of the key pathway component, RELA/p65, revealed that IER3 depletion substantially suppressed both RELA/p65 protein expression and p‐p65 (Ser536) levels. Conversely, IER3 overexpression significantly enhanced RELA/p65 protein expression and p‐p65 (Ser536) levels (Figure 5e). These data suggest that IER3 likely enhances RELA/p65 transcriptional activity through TNF‐*α* upregulation, thereby promoting RELA/p65 protein expression and augmenting Ser536 phosphorylation, which activates the NF‐*κ*B signaling cascade. This mechanism connects the IER3‐positive status to the observed increase in metastatic propensity.

Figure 5Exploring the mechanism of IER3 in colorectal cancer (CRC) cell behavior in vitro. (a) GSEA results using the Hallmark gene sets from MSigDB. (b) Scatter plot of TNF‐*α* expression and IER3 expression. (c) Scatter plot of RELA/p65 expression and IER3 expression. (d) TNF protein levels were measured by ELISA in conditioned medium from tumor cells with knockdown and overexpression of IER3. Data are presented as mean ± SD (SW480: *n* = 3; HCT116: *n* = 3). (e) Western blot results for RELA/p65 and p‐p65 (Ser536). (f) Proliferation curves obtained from the CCK‐8 assay of CRC cells. (g) Colony formation assay of CRC cells with stable IER3 overexpression and IER3 overexpression lines following the addition of PDTC. The bar plot shows colony statistics from the colony formation assay. (h) Transwell assay of CRC cells overexpressing IER3 and stable cell lines following PDTC treatment, viewed at 100× magnification. Bar plot quantifying the number of migrating and invading cells in the Transwell assay for IER3‐overexpressing CRC cells and their stable lines following PDTC treatment. Rescue effects of PDTC treatment. PDTC (10 *μ*M) was maintained continuously in the culture medium for the duration of the experiments.  ^∗^ *p* < 0.05,  ^∗∗^ *p* < 0.01,  ^∗∗∗^ *p* < 0.001, and  ^∗∗∗∗^ *p* < 0.0001.

**Figure:**
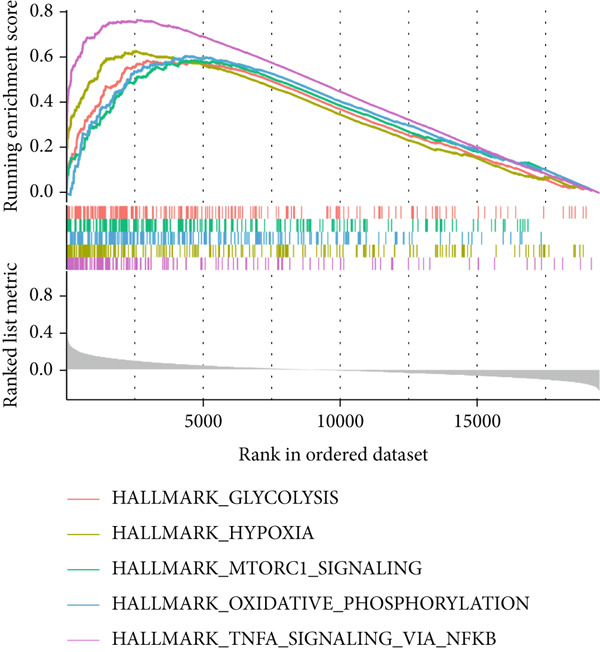
(a)

**Figure:**
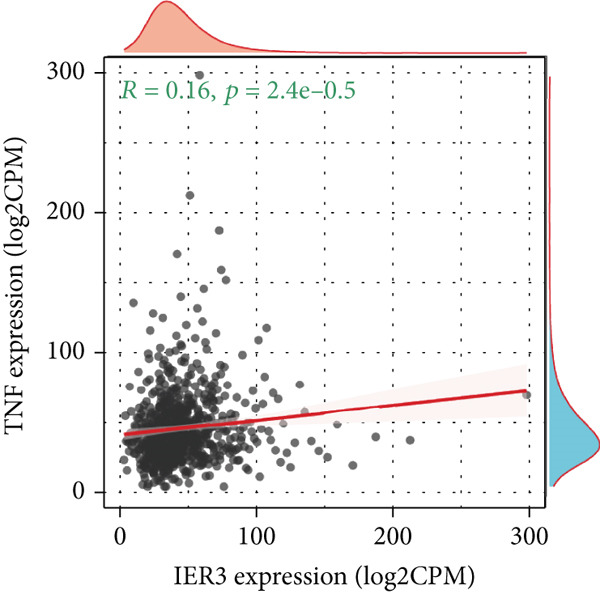
(b)

**Figure:**
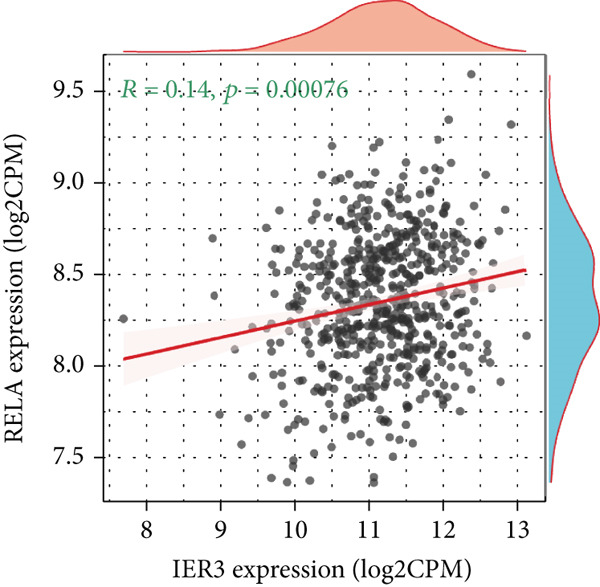
(c)

**Figure:**
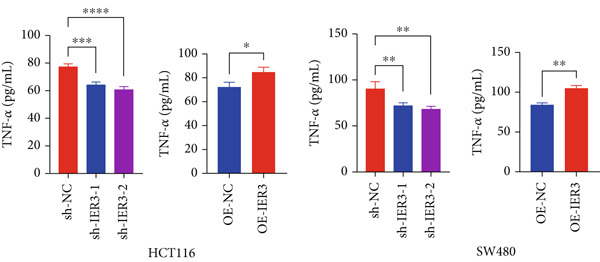
(d)

**Figure:**
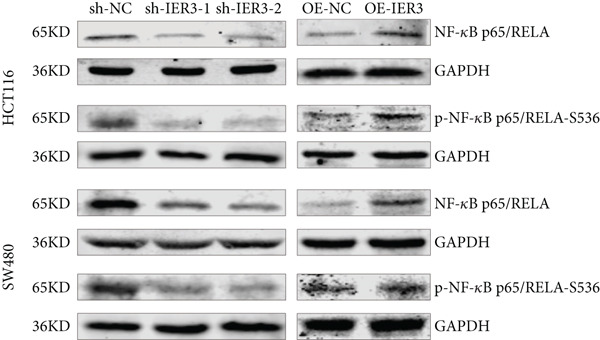
(e)

**Figure:**
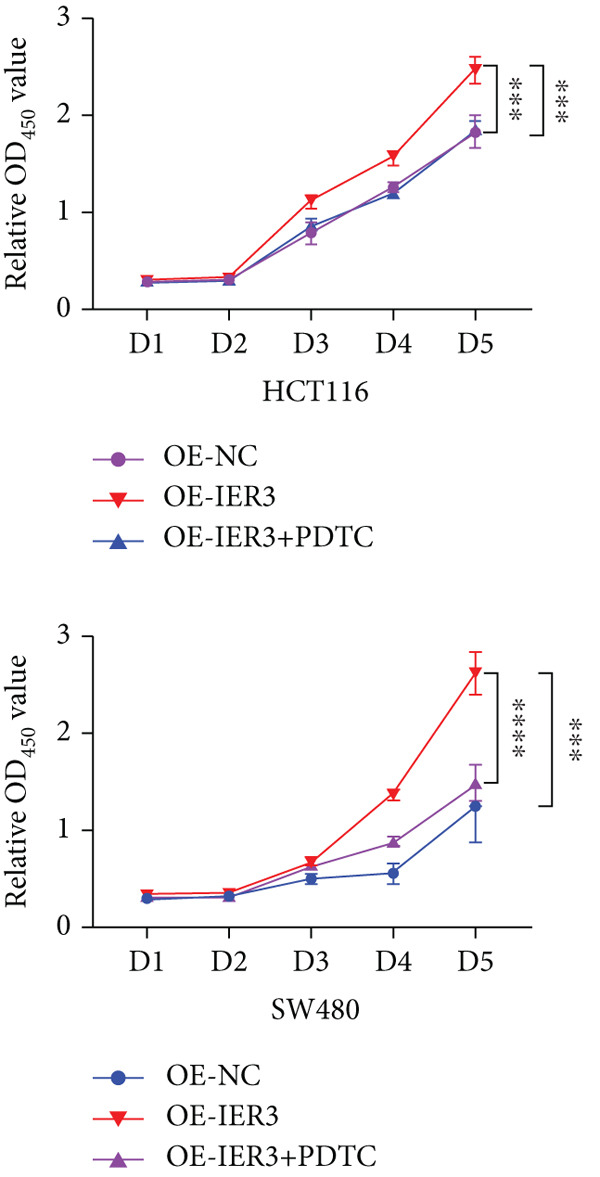
(f)

**Figure:**
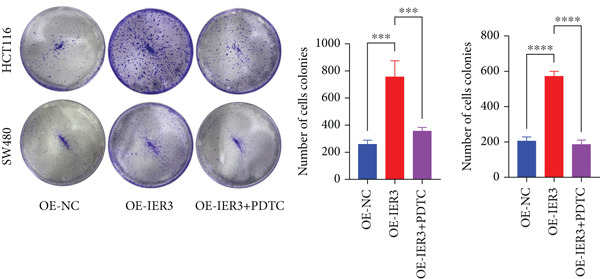
(g)

**Figure:**
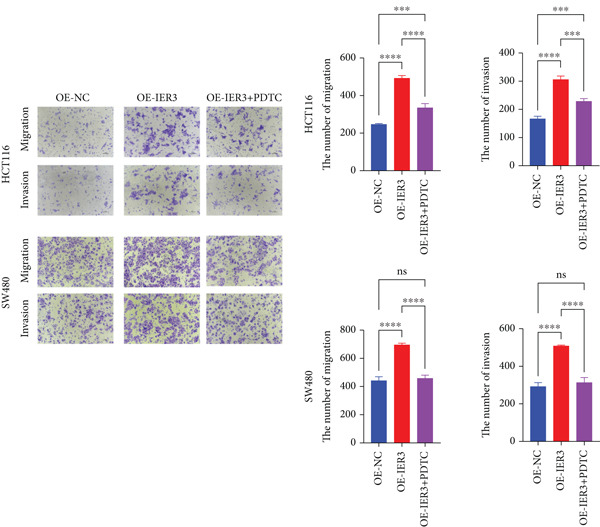
(h)

To determine the dependency of IER3‐driven malignant phenotypes on NF‐*κ*B pathway activity, IER3‐overexpressing CRC cells were treated with the NF‐*κ*B inhibitor PDTC (10 *μ*M). Notably, PDTC abolished the IER3‐induced proliferative advantages in the CCK‐8 and colony formation assays (Figure 5f,g) and similarly negated the enhanced migratory and invasive capacities observed in the Transwell assays (Figure 5h).

These functional rescue data attest that TNF‐*α*/NF‐*κ*B signaling constitutes the principal conduit through which IER3 potentiates tumor progression.

### 3.6. IER3 Expression Modulates Immune Landscape and Dictates Therapeutic Response in CRC

To assess the extent of immune infiltration in IER3‐high versus IER3‐low expressing CRC and to map the immune cell landscape, we employed CIBERSORT for deconvolution analysis.

Violin plots revealed significant differences in the abundance of multiple immune cell types (22 subsets) between the IER3‐high and IER3‐low groups (Figure 6a). Correlation analysis demonstrated that IER3 expression was positively correlated with mast cell abundance but showed significant negative correlations with NK cells, CD8^+^ T cells, and macrophages (Figure 6b,c).

Figure 6IER3 expression modulates immune landscape and dictates therapeutic response in colorectal cancer. (a) Violin plots illustrate the differential abundance analysis of 22 immune cell types between IER3‐high and IER3‐low expressing CRC groups, based on transcriptomic data. (b) Lollipop plots display correlation coefficients between IER3 expression levels and the abundance of 22 immune cell types in CRC, derived from CIBERSORT deconvolution analysis. (c) Scatter plots illustrate the correlation between IER3 expression and the relative abundance of specific immune cell subsets (M2 macrophages, activated mast cells, activated NK cells, CD8^+^T cells, and M1 macrophages). (d) Box plots show predicted responsiveness to immune checkpoint blockade (ICB) therapy based on TIDE scores, stratified by IER3 expression groups (high vs. low). A higher TIDE score indicates poorer predicted ICB responsiveness. (e) Scatter plots demonstrate correlations between IER3 expression levels and the expression of representative immune checkpoint genes (*BTLA*, *CD28*, *CD40LG*, *CD276*, *HAVCR2*, and *TNFRSF4*) in TCGA colorectal cancer samples. (f) Violin plots compare TIDE scores between IER3‐high and IER3‐low expressing colorectal cancer groups. (g) Box plots visualize the estimated sensitivity (IC_50_ values) of IER3‐high and IER3‐low colorectal cancer groups to targeted therapeutic agents (Dasatinib, Ribociclib, ERK_2440, AZD4547, and OF‐1). A lower IC_50_ indicates higher drug sensitivity.

**Figure:**
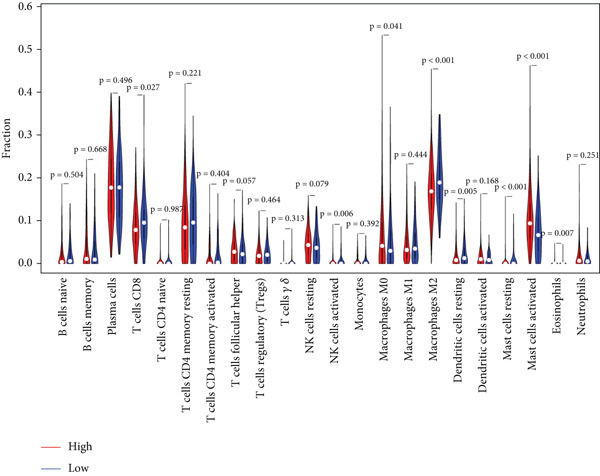
(a)

**Figure:**
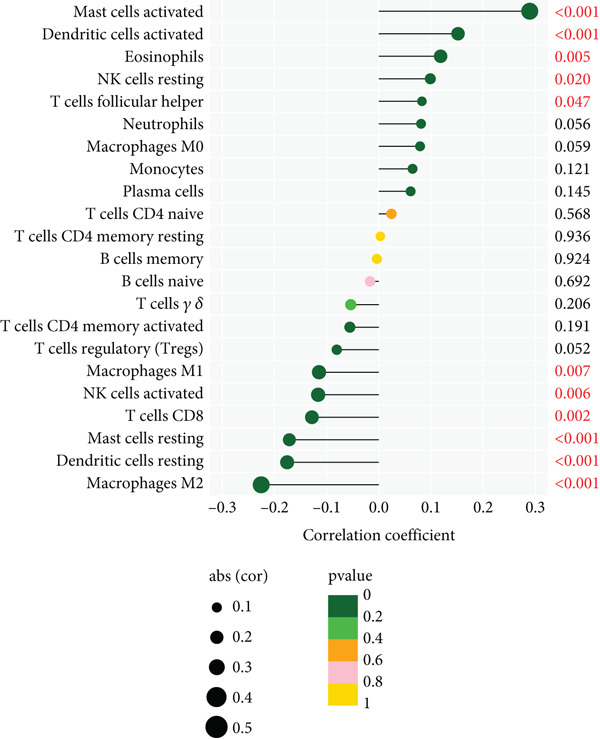
(b)

**Figure:**
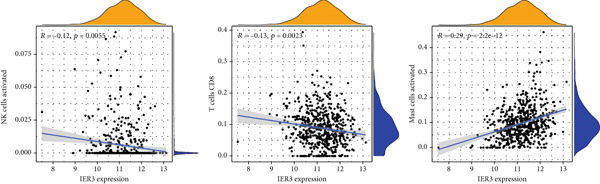
(c)

**Figure:**
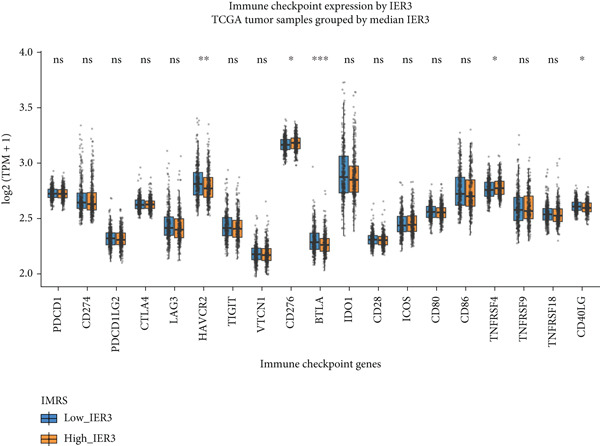
(d)

**Figure:**
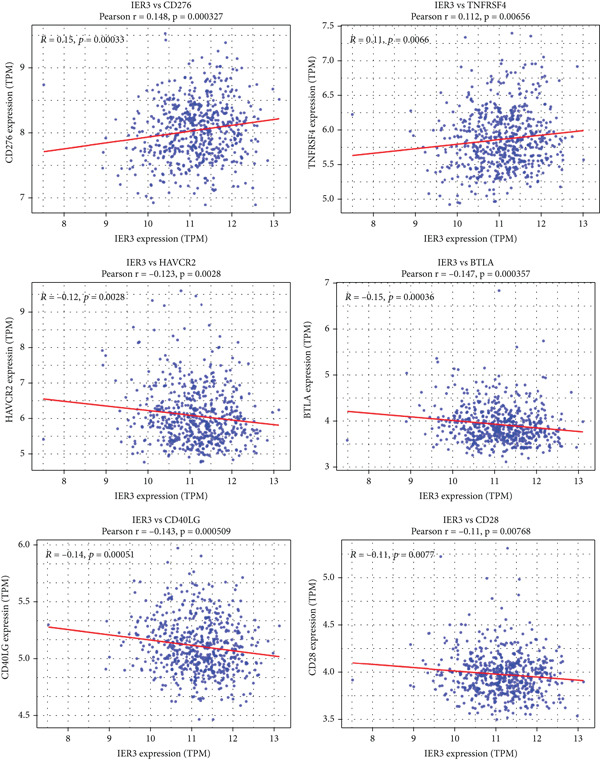
(e)

**Figure:**
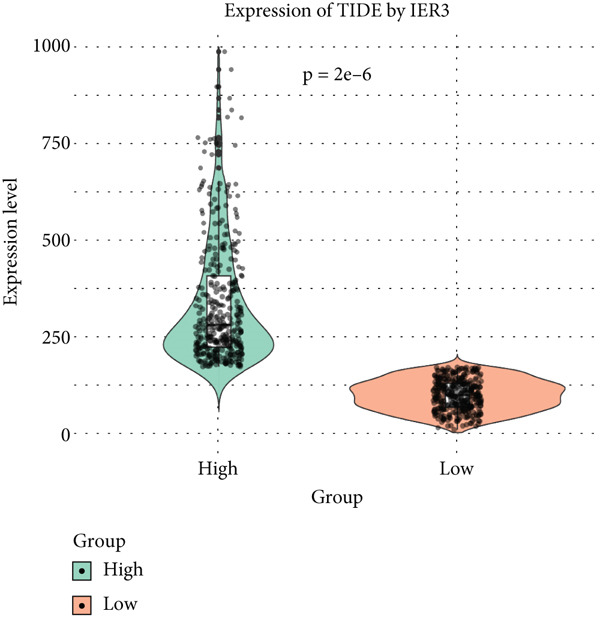
(f)

**Figure:**
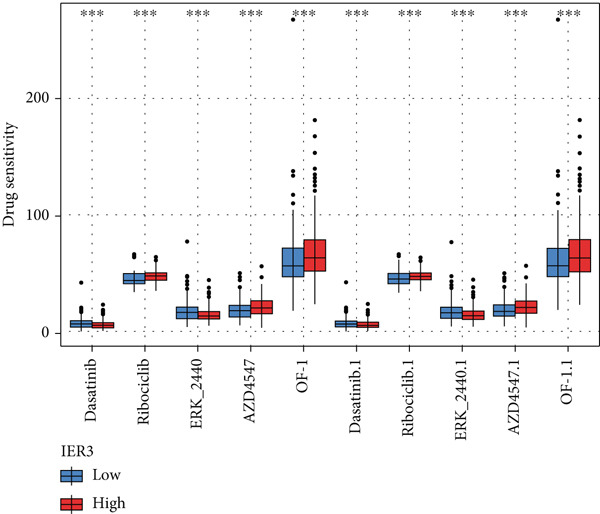
(g)

Analysis of immune checkpoint molecule expression identified two checkpoints (*CD276* and *TNFRSF4*) that were significantly upregulated in the IER3‐high group, whereas three (*HAVCR2*, *BTLA*, and *CD40LG*) were downregulated (Figure 6d). Consistently, IER3 expression exhibited positive correlations with *CD276* and *TNFRSF4* and negative correlations with *HAVCR2*, *BTLA*, *CD40LG*, and *CD28* (Figure 6e).

To predict the response to immunotherapy, we applied the TIDE algorithm. Elevated TIDE scores are associated with diminished efficacy of ICB [23]. Notably, in our study, the IER3‐high group displayed significantly higher TIDE scores than the IER3‐low group (Figure 6f), indicating that high IER3 expression may impede ICB efficacy.

Drug sensitivity profiling using oncoPredict showed that the IER3‐high group exhibited greater sensitivity to six agents (Ribociclib, AZD4547, OF‐1, Ribociclib.1, AZD4547.1, and OF‐1‐1), whereas the IER3‐low group was more sensitive to four drugs (Dasatinib, ERK_2440, Dasatinib.1, and ERK_2440.1) (Figure 6g).

### 3.7. Dominant FN1‐CD44 Axis Mediates IER3^+^ Malignant‐to‐Immune Communication in CRC

To define and understand the complex biological interactions, we employed CellChat to analyze cell–cell communication networks within the CRC microenvironment (Figure 7a). The initial analysis focused on the IER3_High_malignant cell subpopulation as a source of signaling. We observed robust communication probabilities between IER3_High_malignant cells and multiple immune cell types (Figure 7b).

Figure 7Dominant FN1–CD44 axis mediates IER3^+^ malignant‐to‐immune communication in colorectal cancer. (a) Cell–cell interaction counts (A) and interaction weights/strength (B) in colorectal cancer are visualized using circos plots. (b) Communication probabilities from the IER3_High_malignant cell subpopulation (sender) to all other cell types. (c) Heatmaps depict outgoing (A) and incoming (B) signaling patterns associated with cell–cell communication. (d) Heatmap showing the communication probabilities of interactions within the FN1 signaling network. (e) Heatmap displaying centrality scores for signaling pathways related to FN1. (f) Hierarchical plot illustrating the intercellular communication network of FN1 signaling. (g) Bubble plot showing the contribution and interaction probability of each ligand–receptor pair for signaling initiated from the IER3_High_malignant cell population (source) to all target cell populations. (h) Bar plot showing the relative contribution of each ligand–receptor pair to the overall FN1 signaling pathway. (i) Violin plots showing the expression distribution of all genes involved in the relevant signaling pathways across cell populations. (j) FN1 protein levels measured by ELISA in conditioned medium from three groups of tumor cells: control (OE‐NC), IER3‐overexpressing (OE), and IER3‐overexpressing cells treated with the NF‐*κ*B pathway inhibitor PDTC (OE + PDTC). Data are presented as mean ± SD (SW480: *n* = 3; HCT116: *n* = 3).  ^∗^ *p* < 0.05,  ^∗∗^ *p* < 0.01,  ^∗∗∗^ *p* < 0.001, and  ^∗∗∗∗^ *p* < 0.0001; ns, not significant.

**Figure:**
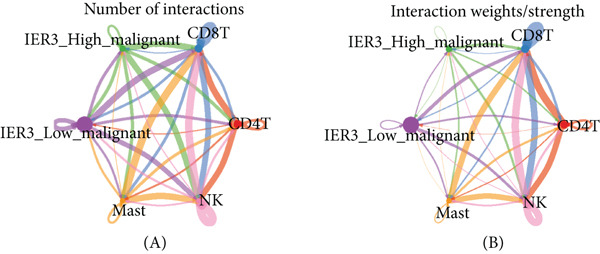
(a)

**Figure:**
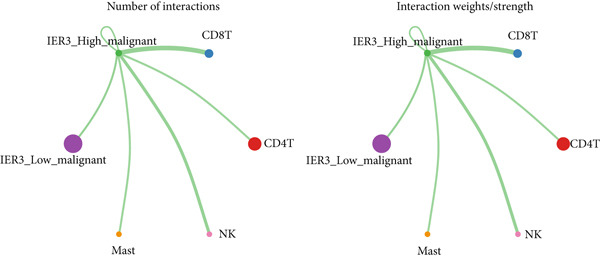
(b)

**Figure:**
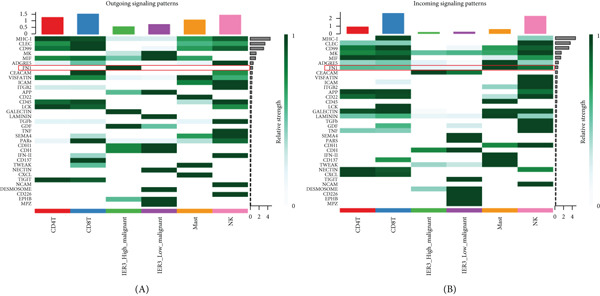
(c)

**Figure:**
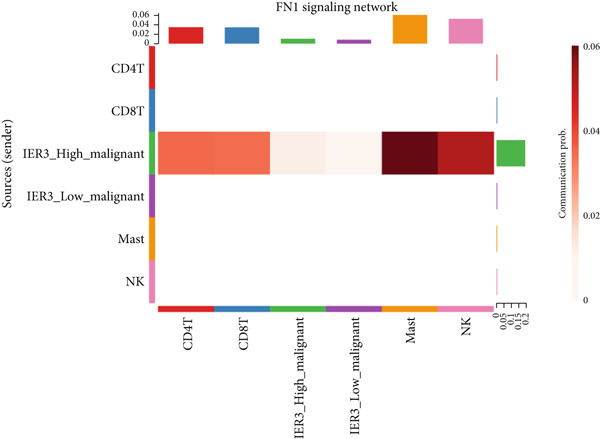
(d)

**Figure:**
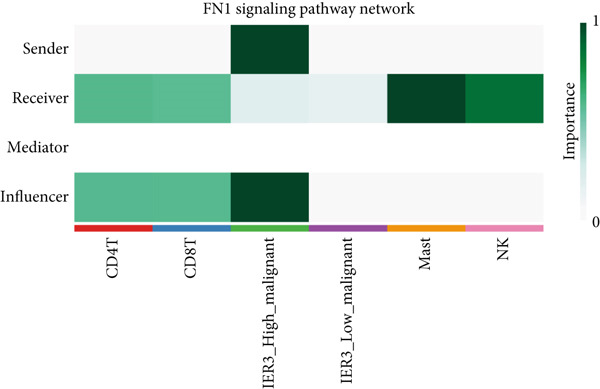
(e)

**Figure:**
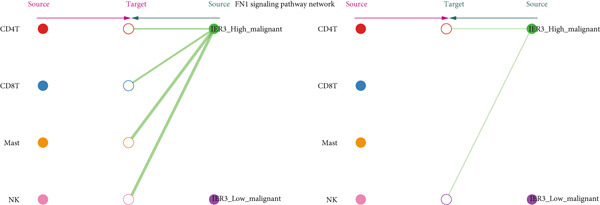
(f)

**Figure:**
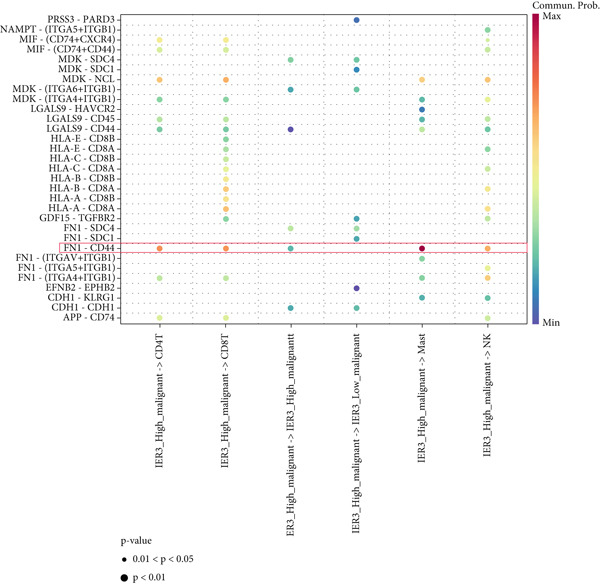
(g)

**Figure:**
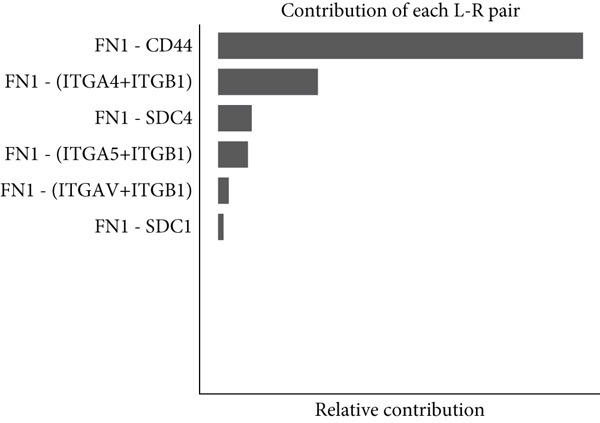
(h)

**Figure:**
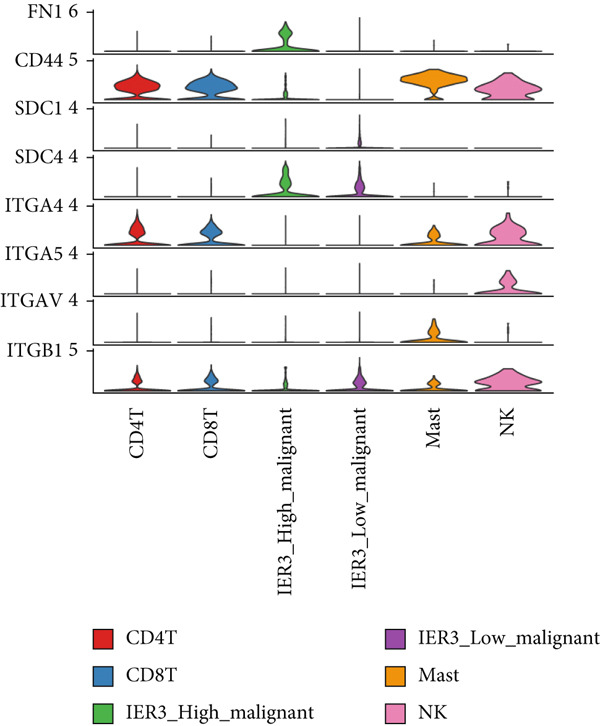
(i)

**Figure:**
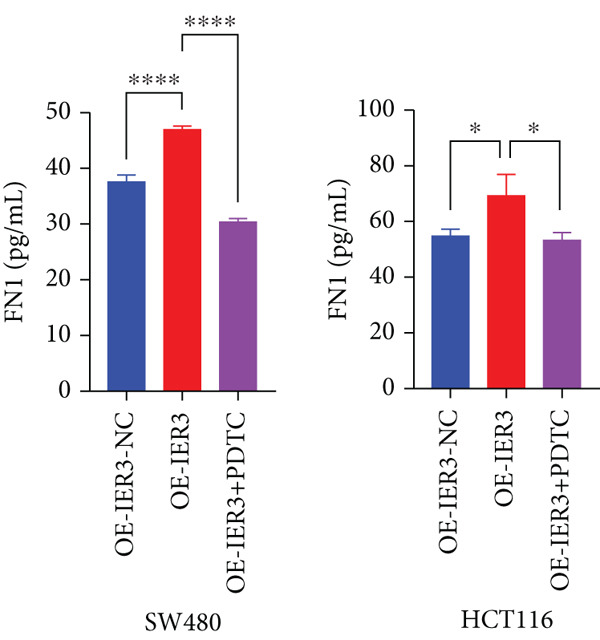
(j)

Subsequent analysis using heatmaps revealed distinct patterns of outgoing and incoming signaling pathways across cell types. The IER3_High_malignant subpopulation exhibited notably high activity in specific outgoing pathways, including FN1, GALECTIN, and GDF signaling (Figure 7c).

Within the FN1 signaling network, IER3_High_malignant cells functioned as the predominant signal senders, with immune cells acting as primary receivers (Figure 7d,e). Hierarchical visualization delineated specific intercellular connections mediated by FN1, identifying prominent signaling interactions originating from IER3_High_malignant cells targeting CD4^+^T cells, CD8^+^T cells, NK cells, and Mast cells (Figure 7f).

To resolve the precise ligand–receptor interactions underlying this communication, we mapped the interactome. Bubble plot analysis demonstrated that signaling from IER3_High_malignant cells to immune cells (CD4^+^ T, CD8^+^ T, NK, and mast) was primarily mediated by the FN1‐CD44 ligand–receptor pair (Figure 7g). The FN1–CD44 axis was the major contributor to the overall strength of the FN1 signaling pathway (Figure 7h). Supporting this, violin plots confirmed that the ligand FN1 was specifically and highly expressed in the IER3_High_malignant subpopulation, whereas the cognate receptor CD44 showed elevated expression in immune cells (Figure 7i).

Finally, functional validation by ELISA demonstrated that IER3 overexpression in tumor cells significantly enhanced FN1 protein secretion in CRC cell lines (SW480 and HCT116). This increase was reversed by cotreatment with the NF‐*κ*B pathway inhibitor PDTC (Figure 7j).

## 4. Discussion

Integrated IHC, single‐cell transcriptomic, functional validation, and clinical correlation analyses elucidated the progression of CRC and identified IER3 as a key driver of CRC advancement through the activation of the TNF‐*α*/NF‐*κ*B signaling pathway. Furthermore, within the tumor immune landscape, IER3^+^ malignant cells remodel the TIME and promote immune evasion via the FN1–CD44 axis, thereby accelerating disease progression. Patients with high IER3 expression exhibited poorer clinical outcomes and reduced responsiveness to ICB.

Through single‐cell transcriptomic profiling, this study characterized an IER3_High malignant subpopulation in CRC. This subpopulation is associated not only with elevated genomic instability, including increased copy number alterations and heightened proliferative capacity, but also with significantly poorer patient prognosis. Trajectory inference analysis indicates that IER3_High malignant cells likely represent a terminal evolutionary state derived from IER3_Low malignant populations, progressively acquired during tumor development. Functionally, this subtype contributes to TIME remodeling and tumor progression through the synergistic activation of multiple oncogenic and inflammatory pathways, including NF‐*κ*B, TNF, and IL‐17 signaling.

We elucidated the mechanistic basis of IER3’s protumorigenic function. The TNF‐*α*/NF‐*κ*B pathway, well established in oncology research, drives colorectal carcinogenesis, promotes invasion, metastasis, and angiogenesis, and induces therapy resistance [31–33]. Our scRNA‐seq data and GSEA of TCGA cohort both indicated that IER3 activates TNF‐*α*/NF‐*κ*B signaling to promote malignant phenotypes in CRC, a finding corroborated by PDTC‐mediated rescue experiments. Regarding tumor immunology, IER3‐high expression was positively correlated with mast cell infiltration but negatively correlated with NK cells, CD8^+^T cells, and macrophages. The FN1–CD44 signaling axis has emerged as the primary mediator of intercellular communication between IER3^+^ malignant cells and immune cells. Activated mast cells can impede T cell activation, induce immune tolerance [14, 34], and secrete proangiogenic factors (e.g., VEGF), accelerating neovascularization and promoting tumor invasion and metastasis [15]. Reduced infiltration and functional impairment of CD8^+^ T cells and NK cells diminish immune surveillance, facilitating immune escape and sustained tumor progression [7, 35]. Furthermore, complex crosstalk between these two core signaling axes, which mediates the protumorigenic and immunosuppressive functions of IER3, is well‐documented, suggesting bidirectional positive regulation that synergistically promotes CRC progression [36–38].

Critically, the role of IER3 in cancer is highly context‐dependent, a duality that underscores the uniqueness of its protumorigenic function in CRC. In certain malignancies, such as some forms of cervical and gastric cancer, IER3 has been reported to act as a tumor suppressor by promoting apoptosis and negatively regulating oncogenic pathways [9, 10, 39, 40]. This functional dichotomy is highly sensitive to tissue specificity, cellular context, and the dynamics of the TME. For instance, in cellular contexts with strong death receptor signaling, IER3 can transiently enhance apoptosis, whereas in environments rich in survival signals like chronic TNF‐*α* exposure—a hallmark of CRC—it can switch to promoting NF‐*κ*B‐mediated survival and inflammation [9, 10]. Our findings demonstrate that in the specific landscape of CRC, characterized by constitutive activation of inflammatory pathways, IER3 is co‐opted into a potent oncogenic driver. The convergence of IER3 expression with the hyperactive TNF‐*α*/NF‐*κ*B pathway and its downstream FN1–CD44 axis creates a feed‐forward loop that robustly promotes malignant progression and immune evasion, thereby clearly defining its protumorigenic role in this cancer type [41, 42]. This context‐specific activation mechanism distinguishes IER3’s function in CRC from its potential suppressive roles in other tissues.

In the context of clinical translation, IER3 expression progressively increases and correlates with adverse clinical and pathological features. Its association with elevated serum CEA levels further supports its potential as a diagnostic and therapeutic biomarker. Moreover, IER3 shows strong utility in predicting responses to ICB and guiding the selection of targeted therapies. These findings highlight IER3 as a promising biomarker for prognosis, treatment stratification, and therapy response prediction in CRC, thereby facilitating personalized treatment and precision oncology.

The critical role of IER3 at the intersection of key signaling pathways and PDTC‐mediated reversal of IER3‐induced FN1 secretion further emphasizes its potential as a therapeutic target for CRC. This study brings IER3 to the forefront of tumor immunotherapy through the TNF‐*α*/NF‐*κ*B pathway–FN1–CD44 axis, which has clinical significance not only in driving tumor progression, but also in providing a potential combined target for overcoming immunotherapy resistance. The interaction between FN1 and CD44 may be directly involved in shaping the immunosuppressive microenvironment, thereby impairing the efficacy of immune checkpoint inhibitors. Therefore, targeting this signal axis combined with anti‐PD‐1/PD‐L1 therapy is expected to produce a synergistic antitumor effect through the dual mechanism of “relieving immunosuppression” and “activating effector molecule attack,” which provides a new strategy for combined immunotherapy of CRC.

Our study has several limitations. First and most critically, our core conclusions on IER3 regulating the immune microenvironment and drug sensitivity are mainly derived from bioinformatics analysis and algorithmic prediction, but these findings are still computational inferences in nature. Ultimately, these important biological hypotheses need to be confirmed by direct functional experiments, such as manipulating the expression of IER3 in cell lines to observe its effect on T cell function, or evaluating the regulatory role of IER3 in the response to immunotherapy and chemotherapy drugs in animal models. Second, the sample size of scRNA‐seq in this study was relatively limited, which may limit the identification of rare cell subsets and the comprehensive characterization of subgroup heterogeneity. In addition, although the clinical tissue sample validation cohort is representative, its size could be scaled up. Based on this, future work should propose validation in larger, ethnically diverse cohorts to assess its generality and clinical applicability. In addition, our exploration at the mechanism level still needs to be deepened. The interaction between IER3 and FN1‐CD44 axis needs further mechanistic elucidation and spatial colocalization verification. At the same time, the potential association between TNF‐*α*/NF‐*κ*B signaling pathway and FN1‐CD44 pathway is also urgent to establish a conclusive causal link through experimental means.

Despite the above limitations, this study provides a new perspective and a solid theoretical framework for understanding the complex function of IER3 in CRC through systematic multiomics analysis. Based on the current findings, future studies should focus on (1) verifying the immunoregulatory and chemosensitivity functions of IER3 in vitro and in vivo, (2) revealing the molecular mechanism of the FN1–CD44 axis downstream of IER3, and (3) rigorously evaluating the therapeutic potential of targeting the NF‐*κ*B pathway, for example using inhibitors such as PDTC, in preclinical animal models, with a view to ultimately translating our findings into effective clinical treatment strategies.

## 5. Conclusions

Our integrated single‐cell transcriptomic, functional, and clinical analyses establish IER3 as a key driver of CRC progression through activation of the TNF‐*α*/NF‐*κ*B signaling pathway and remodeling of the TIME via the FN1–CD44 axis. High IER3 expression identifies a malignant subpopulation associated with aggressive disease, poor prognosis, and diminished response to ICB. These findings underscore the clinical relevance of IER3 as both a biomarker for risk stratification and therapeutic response prediction and a potential target for the development of precision oncology strategies in CRC.

## Ethics Statement

The study was conducted in accordance with the Declaration of Helsinki and approved by the Ethics Committee of the First Hospital of Shanxi Medical University (Approval No.: [2020] Ethical Review K012).

## Consent

Informed consent was obtained from all subjects involved in the study. Written informed consent has been obtained from the patients to publish this paper, if applicable.

## Conflicts of Interest

The authors declare no conflicts of interest.

## Author Contributions

Zg.W.: writing—original draft, data curation, formal analysis, and validation; Y.L.: writing—original draft, validation, and visualization; Yp.Z.: writing—original draft, validation; Q.H.: writing—original draft and resources; J.S.: writing—original draft and validation; Yy.Z.: writing—original draft and validation; Yq.Z.: writing—original draft and visualization; Zm.W.: writing—original draft and resources; C.L.: writing—original draft and formal analysis; Z.L.: writing—review and editing, data curation, and supervision; Y.C.: writing—review and editing, conceptualization, project administration, and supervision.

## Funding

This study was supported by the construction of the National Key Clinical Specialty and the development of Key Medical Disciplines in Shanxi Province.

## Supporting Information

Additional supporting information can be found online in the Supporting Information section.

## Supporting information


**Supporting Information 1** Table S1: Clinicopathological features associated with IER3 expression.

**Supporting Information 2** Figure S1: (a) UMAP visualization of single cells from scRNA‐seq analysis, annotated by color corresponding to distinct sample origins. Cell type identities are demarcated by colored borders for visual distinction. (b) Single‐cell UMAP visualization, with colors representing different tissue regions. (c) UMAP visualization of 23 colored and numbered cell clusters identified by scRNA‐seq. (d) Stacked bar plot showing relative abundance (percentage) of each major cell type in each sample. (e) The bar chart shows the comparison of the proportion of cells among different tissues. (f) UMAP visualization of 17 colored and numbered cell clusters identified by scRNA‐seq. (g) Single‐cell UMAP visualization, with colors representing different tissue regions. (h) InferCNV analysis: chromosomal landscape of inferred copy number variation (CNV) in epithelial cells, colored by CNV level (red for amplification and blue for deletion). (i) The Kaplan–Meier curve showed the overall survival of 610 TCGA CRC patients, classified according to the expression of the IER3_Low_malignant characteristic gene. The *p* value is determined using the log‐rank test. (j) UMAP visualization depicting the distribution of CytoTRACE scores in the malignant cells. Dark green indicates low scores (low stemness), whereas dark red indicates high scores (high stemness).

## Data Availability

The raw data of this study can be publicly accessible in the TCGA and GEO,https://cancergenome.nih.gov/ and https://www.ncbi.nlm.nih.gov/geo/; for more information about GEPIA, please visit https://gepia2.cancer-pku.cn/. The single‐cell transcriptome data are partially sourced from the GEO datasets GSE221575, GSE200997, GSE231559, and GSE161277. All other data supporting the results of this study can be found in the Supporting Information of this article.
