# Supplementary material for: IER3 Promotes Malignant Progression of Colorectal Cancer Through the NF‐κB Pathway
Source: Int J Genomics. 2026 Jan 30;2026:8379666. doi: 10.1155/ijog/8379666 (PMC12859389; doi:10.1155/ijog/8379666)
Supplement: Supplementary file 1 — Supporting Information 1 Table S1: Clinicopathological features associated with IER3 expression. [file IJOG-2026-8379666-s002.docx]

Table 1．Clinicopathological features associated with IER3 expression

| **Characteristics** | n | High expression  (n=42) | Low expression  (n=42) | P-value |
| --- | --- | --- | --- | --- |
| **Age** |  | 64.6±13.55 | 68.81±11.04 | 0.1220 |
| **Genger** |  |  |  |  |
| Male | 47 | 21 | 26 | 0.3795 |
| Female | 37 | 21 | 16 |  |
| **Tumor size** |  |  |  |  |
| **≥5cm** | 33 | 15 | 18 | 0.6553 |
| **＜5cm** | 51 | 27 | 24 |  |
| **T stage** |  |  |  |  |
| Ⅰ-Ⅱ | 6 | 1 | 5 | 0.2016 |
| Ⅲ-Ⅳ | 78 | 41 | 37 |  |
| **N stage** |  |  |  |  |
| N_0_ | 40 | 22 | 18 | 0.5125 |
| N_X_ | 44 | 20 | 24 |  |
| **M stage** |  |  |  |  |
| M_0_ | 58 | 24 | 34 | 0.0326 |
| M_1_ | 26 | 18 | 8 |  |
| **Tumor cell differentiation** |  |  |  |  |
| Poor | 19 | 14 | 5 | 0.0223 |
| Morderate | 50 | 24 | 26 |  |
| well | 15 | 4 | 11 |  |
| **Nerve violation** |  |  |  |  |
| - | 54 | 22 | 32 | 0.0396 |
| + | 30 | 20 | 10 |  |
| **CEA** |  | 9.74（2.07，35.2） | 2.8（1.605，10.93） | 0.0452 |
