# Supplementary figures and images for: IER3 Promotes Malignant Progression of Colorectal Cancer Through the NF‐κB Pathway
Source: Int J Genomics. 2026 Jan 30;2026:8379666. doi: 10.1155/ijog/8379666 (PMC12859389; doi:10.1155/ijog/8379666)

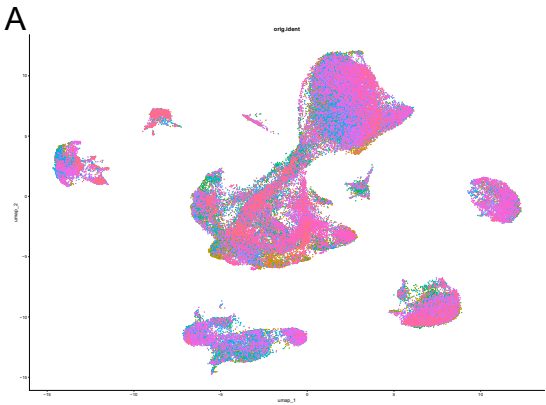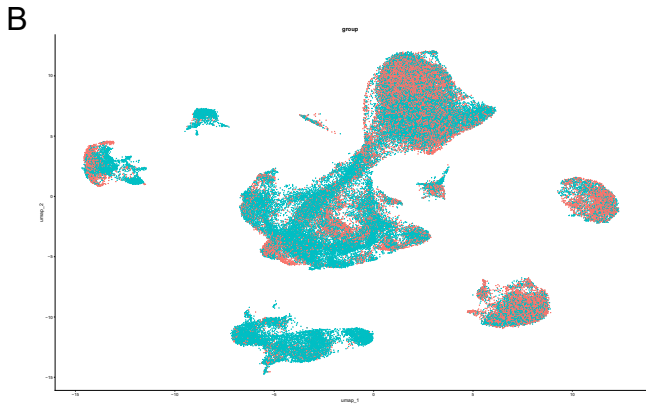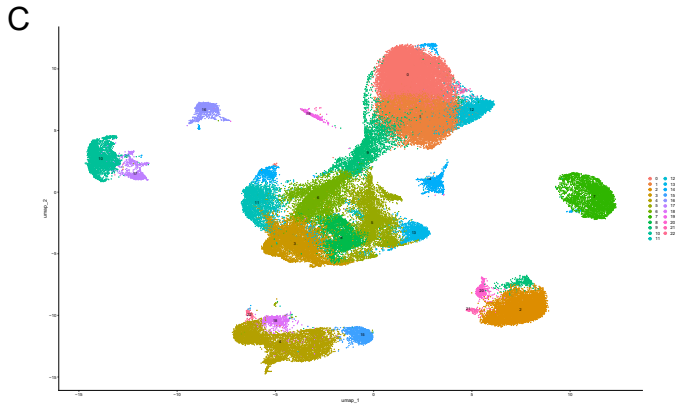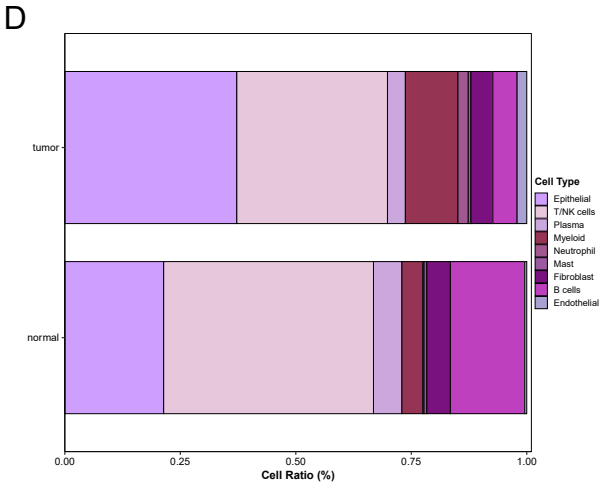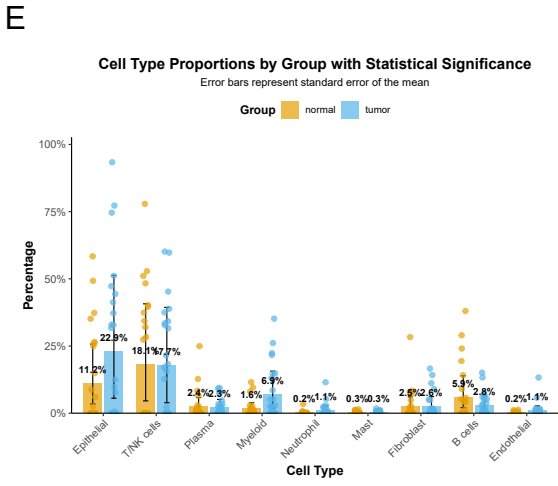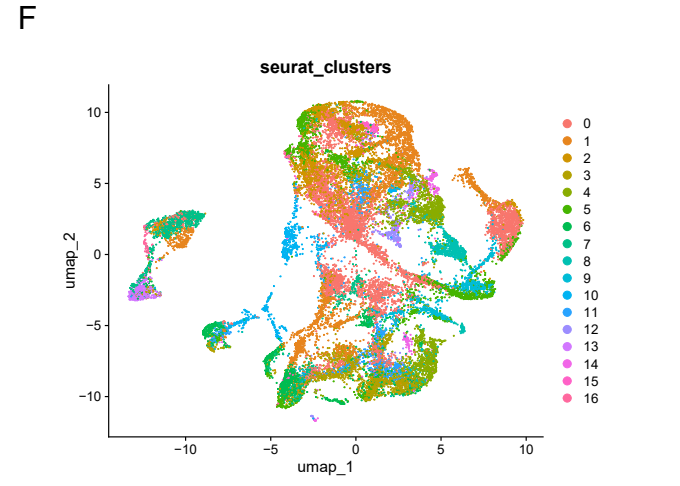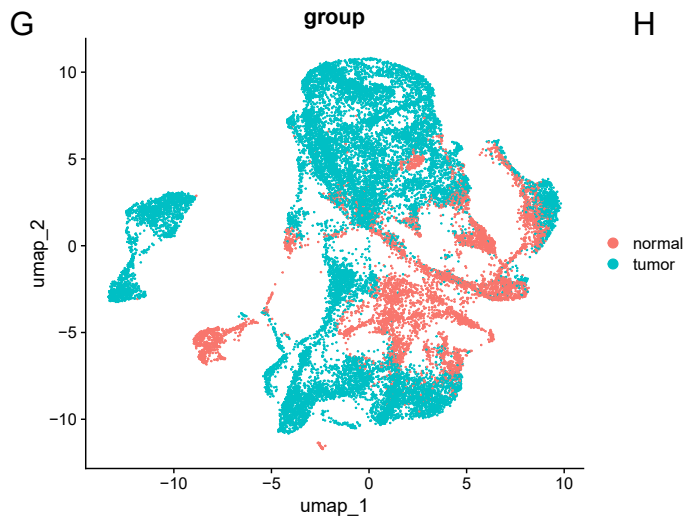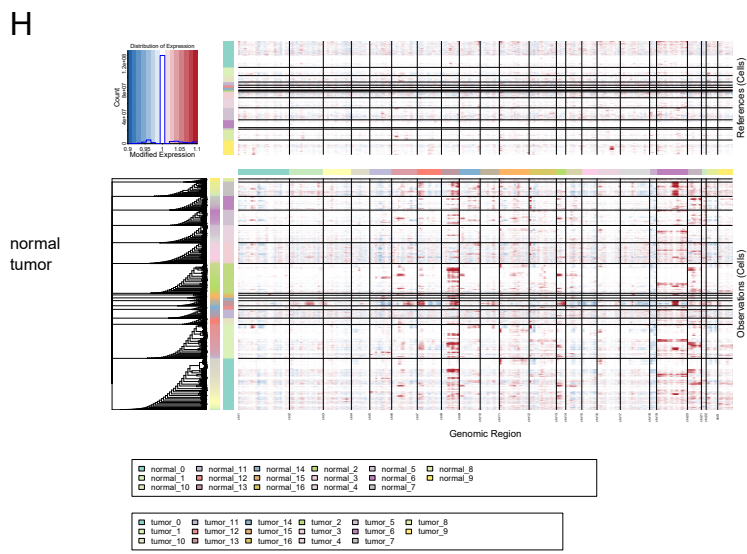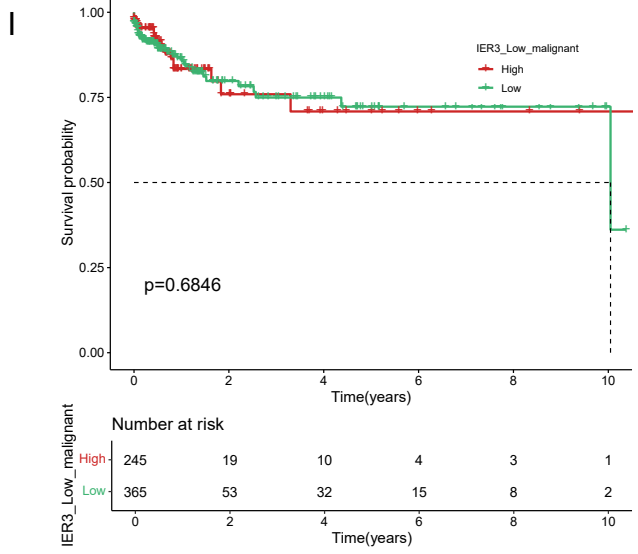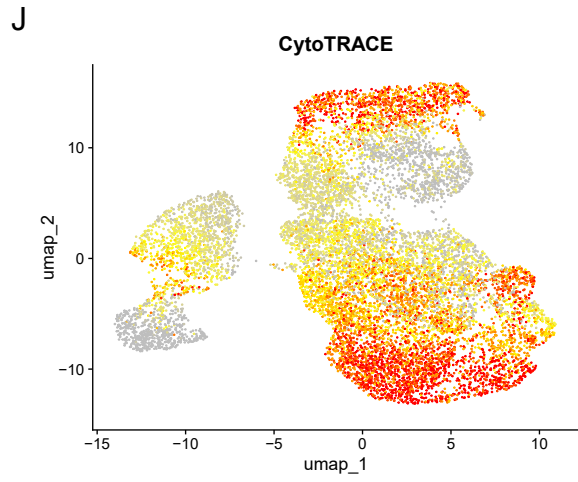

Supplement: Supplementary file 2 — Supporting Information 2 Figure S1: (a) UMAP visualization of single cells from scRNA‐seq analysis, annotated by color corresponding to distinct sample origins. Cell type identities are demarcated by colored borders for visual distinction. (b) Single‐cell UMAP visualization, with colors representing different tissue regions. (c) UMAP visualization of 23 colored and numbered cell clusters identified by scRNA‐seq. (d) Stacked bar plot showing relative abundance (percentage) of each major cell type in each sample. (e) The bar chart shows the comparison of the proportion of cells among different tissues. (f) UMAP visualization of 17 colored and numbered cell clusters identified by scRNA‐seq. (g) Single‐cell UMAP visualization, with colors representing different tissue regions. (h) InferCNV analysis: chromosomal landscape of inferred copy number variation (CNV) in epithelial cells, colored by CNV level (red for amplification and blue for deletion). (i) The Kaplan–Meier curve showed the overall survival of 610 TCGA CRC patients, classified according to the expression of the IER3_Low_malignant characteristic gene. The p value is determined using the log‐rank test. (j) UMAP visualization depicting the distribution of CytoTRACE scores in the malignant cells. Dark green indicates low scores (low stemness), whereas dark red indicates high scores (high stemness). [file IJOG-2026-8379666-s001.pdf]
